# Hyper-Cryptic Marine Meiofauna: Species Complexes in Nemertodermatida

**DOI:** 10.1371/journal.pone.0107688

**Published:** 2014-09-16

**Authors:** Inga Meyer-Wachsmuth, Marco Curini Galletti, Ulf Jondelius

**Affiliations:** 1 Department of Zoology, Swedish Museum of Natural History, Stockholm, Sweden; 2 Department of Zoology, Stockholm University, Stockholm, Sweden; 3 Dipartimento di Scienze della Natura e del Territorio, Università di Sassari, Sassari, Italy; Consiglio Nazionale delle Ricerche (CNR), Italy

## Abstract

Nemertodermatida are microscopically small, benthic marine worms. Specimens of two nominal species, *Sterreria psammicola* and *Nemertinoides elongatus* from 33 locations worldwide were sequenced for three molecular markers. Species delimitation and validation was done using gene trees, haplotype networks and multilocus Bayesian analysis. We found 20 supported species of which nine: *Nemertinoides glandulosum* n.sp., *N. wolfgangi* n.sp., *Sterreria boucheti* n.sp., *S. lundini* n.sp., *S. martindalei* n.sp., *S. monolithes* n.sp., *S. papuensis* n.sp., *S. variabilis* n.sp. and *S. ylvae* n.sp., are described including nucleotide-based diagnoses. The distribution patterns indicate transoceanic dispersal in some of the species. Sympatric species were found in many cases. The high level of cryptic diversity in this meiofauna group implies that marine diversity may be higher than previously estimated.

## Introduction

More than 70% of the earth’s surface is covered by oceans, and sediment covers most of the ocean floor. Marine infauna thus inhabits one of the earth’s largest ecosystems. Sediment meiofauna is a diverse assemblage with representatives from many animal phyla. Despite the vast size of the marine benthic ecosystem, the marine meiofauna is poorly known, and even in well-studied areas numerous undescribed species exist [1]–[4]. Nominal meiofauna species are often reported to have cosmopolitan distributions in concordance with the “Everything is Everywhere (EiE)” hypothesis stating that animals below 1 mm body size are easily dispersed. EiE was originally applied to microorganisms [5] and later extended to organisms up to 1 mm size [6], [7]. However, species identification of meiofauna requires time-consuming microscope studies, which is often only possible when a specialist brings equipment to the field to examine live specimens. Such detailed taxonomic studies have shown a high level of endemicity for some groups, e.g. Platyhelminthes and Acoela [3], thus contradicting the EiE hypothesis; whereas other groups such as gastrotrichs of the genus *Turbanella* seem to conform to a pattern of large distributions [8].

**Figure 1:**
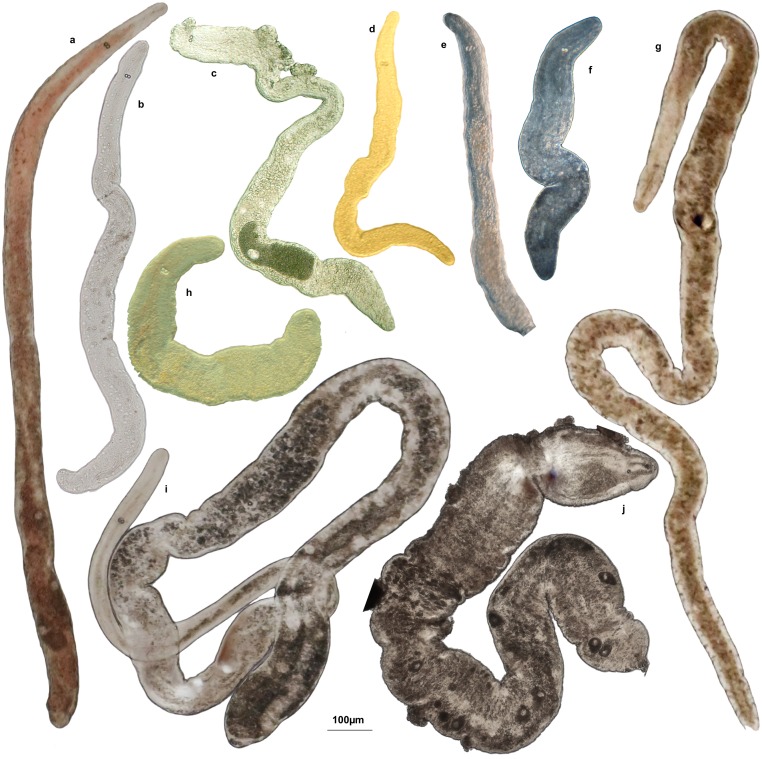
Morphological variation within the genera *Nemertinoides* and *Sterreria*. Light microscope photographs of live specimens in squeeze preparation. a) *Sterreria rubra* from Southern Portugal, b) *S. psammicola* from Southern Portugal, c) *S. martindalei* n.sp. from Waimanolo, Hawaii, d) *S. ylvae* n.sp, from Waimanolo, Hawaii, e) *S. variabilis* n.sp. from New Caledonia, f) *S. variabilis* n.sp. from Bermuda, g) *Nemertinoides elongatus* from Southern Portugal, h) *S. rubra* from Helgoland, North Sea, i) *N. glandulosum* n.sp. from Southern Portugal, j) *N. wolfgangi* n.sp. from Croatia.

The diversity of the marine worms of the taxon Nemertodermatida that are part of the meiofauna in clean sandy sediments was reviewed by Sterrer [9] who recognized eight broadly circumscribed species with a potential for further subdivision as some of them were known only from few specimens, many of which were incomplete. Morphological identification of nemertodermatid species is complicated by the fact that a large number of specimens are juveniles where the diagnostic reproductive organs cannot be studied. In total Sterrer [9] reported that 229 specimens of nemertodermatids were studied by him since 1964. The nominal species with the largest distribution range was *Sterreria psammicola* Sterrer, 1970. Sterrer studied 43 specimens of *S. psammicola* from the North Sea area, the Mediterranean, Caribbean, Australia and Papua New Guinea and considered it “remarkably homogeneous throughout its global distribution range” and regarded *Nemertoderma rubra* (Faubel 1976) [10] as its junior synonym. There is, however, some morphological variation in this cosmopolitan species, most apparent in the pigmentation, which can range from non-existent, with the worms appearing glossy silvery, over a narrow, often only anterior, reddish or brownish “spinal stripe” to a more or less uniform bright red colour (Fig. 1a, h). Pigmented and unpigmented specimens have been recorded from the same site, e.g. around the island of Helgoland, North Sea. The nominal species *Nemertinoides elongatus* Riser, 1987 [11], which is known only from relatively few specimens, is similar in shape to *S. psammicola* and juvenile specimens of the two species cannot be distinguished although adults differ in reproductive anatomy and the morphology and distribution of epidermal gland cells.

While current taxonomy suggests that *Sterreria psammicola* has a cosmopolitan distribution, there are biological factors that indicate limited dispersal ability and consequently a high degree of endemism in these small interstitial marine worms: none of the known nemertodermatid species have dormant eggs or a planktonic stage; small and fragile juveniles hatch from thin-shelled eggs shortly after they have been deposited [12].

A recent estimate of marine eukaryote biodiversity based mainly on expert opinion concluded that there may be 0.7–1.0 million marine species including the 226 000 currently known nominal species. The proportion of cryptic species remaining to be identified was approximated to range between 11% and 43% of the currently known number [13]. Here we follow Bickford *et al.* [14] in regarding as cryptic those species that are or have been classified as the same nominal species due to morphological similarity. Some groups, including Nemertodermatida, were considered too poorly known to allow an estimate of the incidence of cryptic species by Appeltans *et al.* [13]. Costello *et al.* [15] estimated the number of marine species based on the rate of descriptions of new species using data from the World Register of Marine Species (WoRMS) and concluded that there are 300 000 marine species. The latter study did not discuss the effect that undetected cryptic species would have on the estimate. Neither of the two studies defined their concept of species, instead a “legacy species concept” based on the numbers entered into WoRMS was used.

Currently, developments in DNA-sequencing technology and bioinformatics are unleashing the potential for broader and deeper sampling of marine biodiversity. Poorly known meiofauna taxa that may exhibit low morphological complexity or be fragile may thus become better known through metagenetic studies such as those by Fonseca *et al.* [16]. Next-generation sequencing is also likely to reveal additional diversity in the form of cryptic species; see Emerson *et al.* [17] for an example from soil fauna. Metagenetic studies of diversity will be immensely more valuable when a populated database of sequences from known species with revised taxonomy is available.

Here we aim to test the hypothesis that the nominal species *Sterreria psammicola* and *Nemertinoides elongatus* are complexes of cryptic species. Our data is based on 172 specimens that were collected during seven years from 33 different locations (Tab. 1). We sequenced complete or near-complete ribosomal large and small subunit (LSU and SSU) genes and a fragment of the protein coding Histone 3 (H3) gene, and computed separate gene trees under Maximum Likelihood and Bayesian approaches as well as parsimony networks and pairwise distances to generate primary species hypotheses. Clades identified as putative species were tested for genetic isolation using a multilocus Bayesian approach with the software BP&P [18] to generate secondary species hypotheses. Clades with at least three specimens that were supported in at least two of the three gene trees, present as separate haplotype networks under statistical parsimony, had an averaged interspecific pairwise distance at least twice the averaged intraspecific distance, and that were validated by multilocus Bayesian analysis, are formally described and named in this paper. We operate with a species concept in accordance with the “unified species concept” of de Queiroz [19] emphasizing that species are independently evolving lineages that can be diagnosed in a multitude of ways.

**Table 1:** List of localities with geographic coordinates, the number of specimens per gene included in this study.

Country	Locality	Abbr.	Latitude	Longitude	H3	18S	28S	present in clades
**ingroup**								
Bermuda	John Smith’s Bay	Ber	32°19′8.54″N	64°42′39.51″W	4	4	4	*S. variabilis* n.sp.
Croatia	Cherso	CC	45°9′43.26″N	14°17′58.14″E	3	8	8	*N. elongatus*, ***N. wolfgangi*** ** n.sp.**, *S. rubra*, ***S. variabilis*** ** n.sp.**
	Umag	CU	45°25′37″N	13°31′18″E	0	1	1	*S. psammicola*
France	Banyuls-sur-Mer	FB	42°28′53.98″N	3°7′56.55″E	7	8	8	N4, *N. elongatus*, N. *wolfgangi* n.sp., *N. elongatus* n.sp., *S. lundini* n.sp.
Germany	Helgoland; Nordostmauer	GHn	54°11′19.32″N	7°53′6.83″E	3	6	6	*S. rubra*
	Helgoland; Tonne 2	GHt	54°10′48.63″N	7°55′55.49″E	3	4	4	*N. elongatus*, *N. glandulosum* n.sp., *S. lundini* n.sp.
Italy	Acireale	IAc	37°36′13.47″N	15°10′40.56″E	0	1	1	S3
	Agnone	IAg	37°18′37.09″N	15°6′21.03″E	2	2	3	*N. wolfgangi* n.sp., *S. lundini* n.sp.
	Budelli Island	IB	41°17′36.21″N	9°21′39.65″E	0	2	3	*S. rubra*, S. lundini n.sp.
	Castello	ICa	42°45′N	10°52′E	3	3	3	*N. elongatus*, *N. glandulosum* n.sp., *S. lundini* n.sp.
	Formica	IF	42°34′18.48″N	10°53′4.92″E	3	4	5	*S. rubra*
	Ischia	II	40°43′52.06″N	13°57′46.72″E	1	1	1	*S. rubra*
	La Maddalena	IS	41°16′50.88″N	9°19′14.52″E	3	11	11	*S. rubra*, *S. lundini* n.sp., *S. variabilis* n.sp.
	La Maddalena cave	ISc	41°13′30.76″N	9°22′35.36″E	1	2	2	*S. rubra*
	Marcihiaro	IM	42°48′0.4″N	10°44′6.68″E	1	4	4	*S. rubra*, *S. lundini* n.sp., *S. psammicola*
	Miramare	AM	47°42′37.00″N	13°42′43.46″E	0	2	1	*S. rubra, * ***S. psammicola***
	Torre Civette	IC	42°51′17.71″N	10°46′23.56″E	5	16	16	N2, *N. elongatus*, *N. glandulosum* n.sp., *S. rubra*, *S. lundini* n.sp., *S. psammicola*
	Castiglione della Pescaia	IR	42°45′58.98″N	10°51′16.99″E	4	7	7	N1, N3, *N. glandulosum* n.sp., *S. rubra*, ***S. lundini*** ** n.sp.**
	Punta Ala	IW	42°48′24.87″N	10°44′34.37″E	10	15	15	*N. elongatus*, *N. wolfgangi* n.sp., *S. rubra*, *S. lundini* n.sp.
New Caledonia	Amédée	NCA	22°28′39.58″S	166°28′21.54″E	1	1	1	*S. variabilis* n.sp.
	Poe Beach	NCP	21°37′30.72″S	165°23′46.82″E	3	3	2	*S. variabilis* n.sp.
Papua New Guinea	Siar Island	PNGS	05°11′11.94″S	145°48′15.12″E	1	1	1	*S. papuensis* n.sp.
	Tab Island	PNGT	05°10′16.84″S	145°50′18.29″E	1	3	3	*S. papuensis* n.sp.
	Panab Island	PNGP	05°10′18″S	145°48′29″E	6	7	7	*S. papuensis* n.sp., *S. monolithes* n.sp., *S. boucheti* n.sp., P3
	Wanad Island	PNGW	05°08′07″S	145°49′16″E	11	13	13	***S. papuensis*** ** n.sp.**, S7, ***S. monolithes n.sp.***, ***S. boucheti*** ** n.sp.**, *S. variabilis* n.sp.
Portugal	Faro	PF	36°57′32.1″N	W7°57′3.78″E	9	11	12	*N. elongatus*, ***N. glandulosum*** ** n.sp.**, *S. rubra*, S2, *S. psammicola*, *S. variabilis* n.sp.
	Ilha da Culatra	PC	36°58′55.2″N	7°52′1.2″E	2	3	3	*S. rubra*
Sweden	Grisbådarna	SG	58°55′22.15″N	10°49′48.79″E	2	3	3	*N. elongatus*, *S. rubra*
	Kalkgrund	SK	58°55′22.94″N	11°2′42.86″E	4	5	5	*N. elongatus*, S2
USA, Hawaii	Waimanalo	H	21°19′35.68″N	157°40′57.93″W	8	8	8	***S. martindalei*** ** n.sp.**, ***S. ylvae*** ** n.sp.**, *S. variabilis* n.sp.
**outgroup**								
Norway	Raunefjord	N	60°16′15.6″N	5°10′51.6″E	1	3	3	*Meara stichopi*
Sweden	Grisbådarna	SG	58°55′22.15″N	10°49′48.79″E	2	2	2	*Nemertoderma westbladi*
	Södra Hällsö	SH	58°56′41.68″N	11°4′57.14″E	1	1	1	*N. westbladi*
	Lilleskärslätten	SL	58°52′55.63″N	11°6′34.63″E	1	1	1	*N. westbladi*
Sum					106	166	168	*N. westbladi*

Species or clades collected at a given locality are shown with type localities for a given species shown in bold. N abbreviate clades belonging to the genus *Nemertinoides* and S indicates those belonging to the genus *Sterreria*.

## Materials and Methods

### Permits

Taxa used in this study are interstitial invertebrates, which do not need special sampling permits, as they are not subject to regulations of species protection and are collected within small amounts of sediment.

For sampling around Helgoland, Germany, at Waimanolo, Hawaii, in Norway, Sweden and most of the Mediterranean, no specific or additional sampling permits for the collection of small amounts of marine sediments were required. Geographic coordinates for each site are given in table 1 of the manuscript. A sampling permit for Bermuda was granted by the Department of Conservation Services, Bermuda; the permit for New Caledonia by the Direction de l’environnement, Nouvelle Calédonie. The permit for sampling in the Parco Nazionale dell'Arcipelago di La Maddalena, Sardinia, was granted by the National Park authority. Sampling in Papua New Guinea took place under a permit delivered by the Papua New Guinea Department of Environment and Conservation.

### Specimens

Specimens were extracted from sediments using isotonic magnesium chloride solution [20] and identified under a dissecting microscope sometimes in combination with a compound microscope. Specimens were photographed using a compound microscope, if possible equipped with differential interference contrast optics, before fixing in ethanol or RNAlater. Their microscopic size necessitates use of whole specimens for DNA extraction. To ensure a direct link between morphology and gene sequences all type specimens were photographed prior to preservation for DNA extraction and images are deposited as illustrations of the type material, see table 2 for museum and genbank accession numbers. For the description of the position of morphological characters, a relative scale (U) is used with the anterior tip of the animal corresponding to 0 U and the posterior tip to 100 U [21]. Measurements, however, are difficult to take as animals seldom lie straight and relaxed for a sufficiently long time and in many cases specimens are incomplete, as the worms are fragile.

**Table 2:** List of all individuals used in this study sorted by clade, with Zoobank Life Science Identifiers (LSID) where applicable, connecting collection code (used in the scratchpads database for Acoela and Nemertodermatida at http://acoela.myspecies.info/), genbank accession numbers per gene and the museum collection numbers for type material.

species/clade	ZooBank LSID	collectioncode	SMNHtypenumber	Genbank accession number		
				LSU	SSU	H3
*N. elongatus*		07–010		KM062712	KM062546	KM194610
		07–011		KM062713	KM062547	KM194611
		07–013		KM062714	KM062548	KM194612
		07–030		KM062716	KM062550	KM194614
		07–040		KM062719	KM062553	KM194616
		07–051		KM062720	KM062554	KM194617
		07–074		KM062722	KM062556	KM194618
		07–076		KM062723	KM062557	KM194619
		07–078		KM062724	KM062558	KM194620
		08–090		KM062728	KM062563	
		08–110		KM062740	KM062575	
		08–120		KM062745	KM062580	
		09–001		KM062749	KM062584	KM194635
		11–143		KM062799	KM062633	KM194665
		13–170		KM062814	KM062648	KM194676
		13–176		KM062815		
		13–180		KM062816	KM062649	KM194677
		13–441		KM062824	KM062657	
		13–442		KM062825	KM062658	KM194683
		13–446		KM062826	KM062659	
		MCG04		KM062834		KM194684
*N. glandulosum* n.sp.	urn:lsid:zoobank.org:act:DFBD9E91-83E2-4567-91ED-BF279F16C824	07–001		KM062705	KM062539	KM194607
		07–002		KM062706	KM062540	KM194608
		07–003		KM062707	KM062541	
		07–007		KM062709	KM062543	KM194609
		08–115		KM062741	KM062576	KM194632
		08–122		KM062747	KM062582	KM194634
		11–046		KM062792	KM062626	KM194660
		11–071		KM062793	KM062627	KM194661
		13–181		KM062817	KM062650	KM194678
		13–185	8631	KM062819	KM062652	KM194679
		MCG05		KM062835	KM062668	KM194685
		MCG07		KM062837	KM062670	KM194687
		MCG08		KM062838	KM062671	KM194688
*N. wolfgangi* n.sp.	urn:lsid:zoobank.org:act:1CC4C7FC-5CAD-4DD0-9C0E-039390D11356	09–041		KM062757		KM194640
		08–095		KM062732	KM062567	KM194627
		08–096		KM062733	KM062568	KM194628
		08–109		KM062739	KM062574	KM194631
		09–058		KM062763	KM062597	KM194642
		13–453	8632	KM062828	KM062661	
		MCG10		KM062840	KM062673	KM194690
		MCG13		KM062843	KM062676	KM194691
		MCG15		KM062845		KM194692
N1		08–102		KM062736	KM062571	
N2		08–121		KM062746	KM062581	
		08–123		KM062748	KM062583	
N3		08–098		KM062734	KM062569	KM194629
		08–100		KM062735	KM062570	KM194630
N4		MCG06		KM062836	KM062669	KM194686
N4		MCG09		KM062839	KM062672	KM194689
P3		PNG60		KM062858	KM062690	KM194699
		PNG61				KM194700
*S. boucheti* n.sp.	urn:lsid:zoobank.org:act:65760DAD-F39F-4B29-9539-F091D45774FA	PNG70		KM062863	KM062695	KM194705
		PNG54		KM062854	KM062686	KM194697
		PNG68		KM062862	KM062694	KM194704
		PNG72		KM062864	KM062696	
		PNG75	8633	KM062866	KM062698	KM194707
		PNG83		KM062869	KM062701	KM194709
		PNG87		KM062872	KM062704	KM194712
*S. lundini* n.sp.	urn:lsid:zoobank.org:act:F05F5C93-D3C5-4AEA-969D-F1AD2ADE8C20	08–093		KM062730	KM062565	KM194626
		08–094		KM062731	KM062566	
		08–117	8634	KM062743	KM062578	KM194633
		09–013		KM062753	KM062588	
		09–035		KM062756	KM062591	KM194639
		09–053		KM062761	KM062595	KM194641
		10–076		KM062779	KM062613	
		10–110		KM062784	KM062618	
		11–073		KM062794	KM062628	
		MCG01		KM062831	KM062665	
		MCG03		KM062833	KM062667	
		MCG11		KM062841	KM062674	
		MCG14		KM062844	KM062677	
*S. martindalei* n.sp.	urn:lsid:zoobank.org:act:AD07EBF4-F151-4139-A3FC-8BB548E4E8D6	10–055		KM062771	KM062605	KM194648
		10–056	8635	KM062772	KM062606	KM194649
		10–060		KM062774	KM062608	KM194651
*S. monolithes* n.sp.	urn:lsid:zoobank.org:act:638DA2C2-4120-4270-8442-C8D857ED78F6	PNG57		KM062856	KM062688	KM194698
		PNG84	8636	KM062870	KM062702	KM194710
		PNG85		KM062871	KM062703	KM194711
*S. papuensis* n.sp.	urn:lsid:zoobank.org:act:B5470A6B-3FBF-432A-84A0-5B980EB9469A	PNG48		KM062849	KM062681	KM194694
		PNG49		KM062850	KM062682	
		PNG50		KM062851	KM062683	KM194695
		PNG51		KM062852	KM062684	
		PNG52		KM062853	KM062685	KM194696
		PNG56		KM062855	KM062687	
		PNG58		KM062857	KM062689	
		PNG62		KM062859	KM062691	KM194701
		PNG66		KM062860	KM062692	KM194702
		PNG77	8637	KM062868	KM062700	
*S. psammicola*		07–006		KM062708	KM062542	
		09–012		KM062752	KM062587	
		13–155		KM062810	KM062644	KM194673
		13–186		KM062820	KM062653	KM194680
		13–483		KM062829	KM062662	
		13–508	8640	KM062830	KM062663	
*S. rubra*		07–008		KM062710	KM062544	
		07–009		KM062711	KM062545	
		07–031		KM062717	KM062551	
		08–092		KM062729	KM062564	KM194625
		08–103		KM062737	KM062572	
		08–116		KM062742	KM062577	
		08–118		KM062744	KM062579	
		09–002		KM062750	KM062585	KM194636
		09–005		KM062751	KM062586	KM194637
		09–028		KM062754	KM062589	KM194638
		09–029		KM062755	KM062590	
		09–049		KM062758	KM062592	
		09–051		KM062759	KM062593	
		09–052		KM062760	KM062594	
		09–054		KM062762	KM062596	
		09–059		KM062764	KM062598	
		09–060		KM062765	KM062599	KM194643
		09–061		KM062766	KM062600	
		10–073		KM062776	KM062610	
		10–074		KM062777	KM062611	
		10–075		KM062778	KM062612	KM194653
		10–090		KM062780	KM062614	
		10–092		KM062781	KM062615	KM194654
		10–093		KM062782	KM062616	
		10–098		KM062783	KM062617	
		10–117		KM062785	KM062619	
		10–184		KM062787	KM062621	KM194656
		10–188		KM062788	KM062622	
		10–247		KM062789	KM062623	KM194657
		11–139		KM062795	KM062629	KM194662
		11–140		KM062796	KM062630	KM194663
		11–141		KM062797	KM062631	KM194664
		11–142		KM062798	KM062632	
		11–144		KM062800	KM062634	
		11–184		KM062801	KM062635	
		13–094		KM062806	KM062640	KM194670
		13–096		KM062807	KM062641	KM194671
		13–097		KM062808	KM062642	
		13–148		KM062809	KM062643	KM194672
		13–158		KM062813	KM062647	
		13–182		KM062818	KM062651	
		13–429		KM062822	KM062655	KM194681
		13–431		KM062823	KM062656	KM194682
		13–512			KM062664	
		MCG02		KM062832	KM062666	
*S. variabilis* n.sp.	urn:lsid:zoobank.org:act:FF59FF43-B445-46E0-A721-9DF8950D9B38	08–055		KM062725	KM062559	KM194621
		08–056			KM062560	KM194622
		08–061		KM062726	KM062561	KM194623
		08–078		KM062727	KM062562	KM194624
		09–063		KM062767	KM062601	KM194644
		10–053		KM062769	KM062603	KM194646
		10–154		KM062786	KM062620	KM194655
		11–416		KM062802	KM062636	KM194666
		11–418		KM062803	KM062637	KM194667
		11–424		KM062804	KM062638	KM194668
		11–425		KM062805	KM062639	KM194669
		13–156		KM062811	KM062645	KM194674
		13–428		KM062821	KM062654	
		13–452	8638	KM062827	KM062660	
		PNG74		KM062865	KM062697	KM194706
		PNG76		KM062867	KM062699	KM194708
*S. ylvae* n.sp.	urn:lsid:zoobank.org:act:737BC224-D056-458D-B33B-AC564F6C7499	10–043		KM062768	KM062602	KM194645
		10–054	8639	KM062770	KM062604	KM194647
		10–058		KM062773	KM062607	KM194650
		10–064		KM062775	KM062609	KM194652
S2		07–072		KM062721	KM062555	
		08–104		KM062738	KM062573	
		13–157		KM062812	KM062646	KM194675
S3		MCG12		KM062842	KM062675	
S7		PNG67		KM062861	KM062693	KM194703
*M. stichopi*		Meara_a		KM062846	KM062678	KM194693
		Meara_c		KM062847	KM062679	
		Meara_e		KM062848	KM062680	
*N. westbladi*		07–028		KM062715	KM062549	KM194613
		07–035		KM062718	KM062552	KM194615
		10–255		KM062790	KM062624	KM194658
		10–317		KM062791	KM062625	KM194659

N. abbreviates the genus *Nemertinoides*, S. the genus *Sterreria*, abbreviations with numbers indicate putative species per genus not formally described in this paper. Type material is deposited at the Swedish Museum of Natural History (SMNH) in Stockholm, Sweden.

### DNA extraction, amplification and sequencing

DNA was extracted using the Qiagen Micro Tissue Kit. The microscopic size and corresponding low yield of extracted DNA from the specimens as well as the unavailability of prior sequence data severely limited the choice of nucleotide markers. We were able to consistently amplify and sequence rRNA genes as well as the nuclear protein coding Histone 3 gene. The large ribosomal subunit gene was obtained from 168 specimens with an alignment length of 3583 bp, the small ribosomal subunit gene from 166 specimens (1792 bp) and H3 from 106 specimens (328 bp). All markers were amplified and sequenced using several different primer combinations (Tab. 3), and, in the case of SSU, a nested PCR approach.

**Table 3:** Primers used in this study for sequencing of SSU, LSU and H3.

Gene	Name	sequence	direction
SSU	TimA[65]	AMCTGGTTGATCCTGCCAG	forward
	TimB[65]	TGATCCATCTGCAGGTTCACCT	reverse
	S30[66]	GCTTGTCTCAAAGATTAAGCC	forward
	5FK[65]	TTCTTGGCAAATGCTTTCGC	reverse
	4FB[65]	CCAGCAGCCGCGGTAATTCCAG	forward
	1806R[66]	CCTTGTTACGACTTTTACTTCCTC	reverse
LSU	U178[67]	GCACCCGCTGAAYTTAAG	forward
	L1642[67]	CCAGCGCCATCCATTTTCA	reverse
	1200F[67]	CCCGAAAGATGGTGAACTATGC	forward
	R2450[67]	GCTTTGTTTTAATTAGACAGTCGGA	reverse
	UJ2176[68]	TAAGGGAAGTCGGCAAATTAGATCCG	forward
	L3449[67]	ATTCTGACTTAGAGGCGTTCA	reverse
	U1846[67]	AGGCCGAAGTGGAGAAGG	forward
	L2984[67]	CTGAGCTCGCCTTAGGACACCT	reverse
	28SP1F5Ster	CTGAGAAGGGTGTGAGACCCGTAC	forward
	28SP1R1Ster	TCCCGTAGATCCGATGAGCGTC	reverse
H3	H3 AF[69]	ATGGCTCGTACCAAGCAGACVGC	forward
	H3 AR[69]	ATATCCTTRGGCATRATRGTGAC	reverse
	H3FNem	ATGGCTCGTACCAAGCAGACG	forward
	H3RNem	GTCACCATCATGCCCAAGGA	reverse

TimA and TimB are outer primers spanning the length of the whole fragment. S30 and 5FK are internal primer for the first part and 4FB and 1806R for the second part. H3FNem and HRNem are the Colgan *et al.* [69] primers modified for Nemertodermatidae.

Sequence editing, alignment (MAFFT [22]), translation into amino acids and checks for open reading frames were performed using the Geneious Pro 7.0.4. software package created by Biomatters available from http://www.geneious.com. The alignments were tested for random similarity with the program Aliscore [23], [24] using the default settings. jModeltest v. 2.1.1. [25] analyses were performed for each dataset in order to test the datasets for the use of the proportion of invariable sites (I, propinvar) and the rate variation across sites (G) and to obtain values to set useful priors. Evolutionary neutrality of the coding gene H3 was tested using Tajima’s D calculated with the software MEGA 5 [26]. Saturation of the H3 gene was detected through plotting the uncorrected p-distances versus the phylogenetic distance using an R-script [27].

We chose two other nemertodermatid species, *Nemertoderma westbladi*, Steinböck 1930 and *Meara stichopi*, Westblad 1949 as outgroup taxa.

### Phylogenetic “species discovery”

The Geneious package (v. 7.0.4.) was also used to calculate pairwise distances between sequences within and between putative species. For this the LSU and SSU alignments were trimmed by eye to 2009 bp and 1502 bp respectively in order to have sequences of similar lengths but keep most of the information. Those specimens represented by less than half of the alignment length were excluded (s. Supplementary table ST1 for details).

Parsimony haplotype networks were computed using the software TCS 1.21 [28] with the reduced and trimmed datasets for LSU (further reduced to 154 specimens and 2009 bp) and SSU. Gaps were considered a fifth state. For relatively fast evolving mitochondrial genes, a 95% threshold has been shown to recover known species reliably [29]. To account for a slower evolutionary rate the connection limit was set to 98% for the LSU and SSU genes; an additional analysis with the connection limit of 90% was performed for the higher resolving Histone 3 dataset.

Maximum Likelihood (ML) and bootstrap support calculations were performed by raxmlGUI [30] using the GTR+G+I evolutionary model and the rapid bootstrap algorithm with 1000 bootstrap reiterations.

Bayesian analyses were performed using the program MrBayes 3.2.1. [31]. No evolutionary model was set and the program was allowed to sample the entire model space of the GTR model by defining nst = mixed. The proportion of invariable sites and G were applied with the prior set to shapepr = Uniform(0.05,1.00) for SSU and LSU and shapepr = Uniform(0.05,2.00) for H3; the pinvarpr was left at the default. Analyses were stopped when the standard deviation of split frequencies was between 0.01 and 0.05, indicating sufficient convergence and a relative burn-in of 25% was used. No concatenated analyses for all three genes combined were conducted. This would conceal incongruences between the gene trees and therefore possibly lead to subsequent errors in the validation of species using BP&P [32].

Trees were visualized using FigTree v1.3.1. [33]. Alignments and tree-files are deposited with Treebase (http://purl.org/phylo/treebase/phylows/study/TB2:S15809).

### “Species” validation

**Figure 2:**
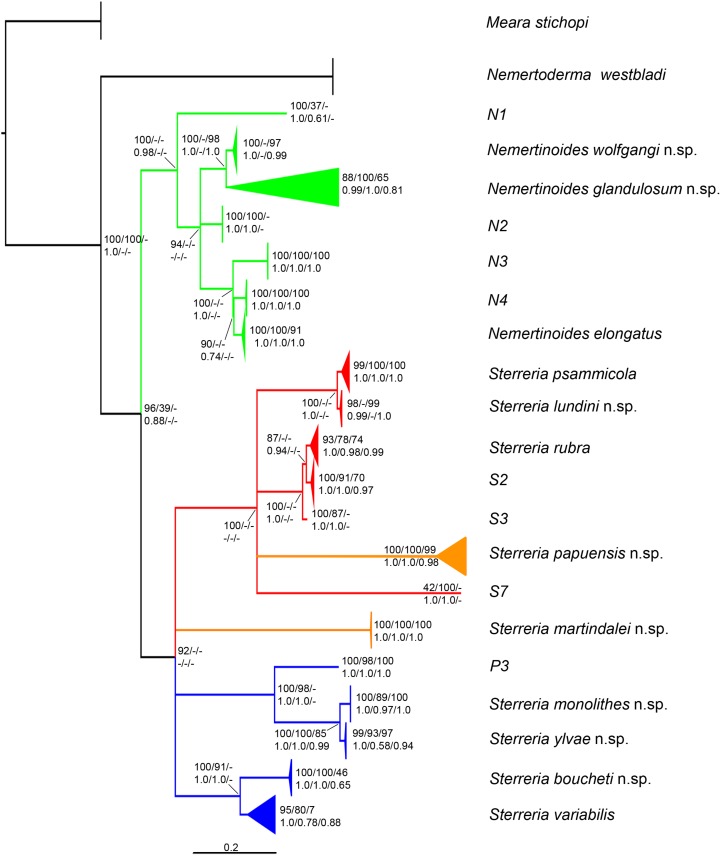
Majority rule consensus tree (75%) of the LSU ML tree with collapsed terminals. The colours correspond to partitions for BP&P analyses, green indicates the *Nemertinoides* group, red the mainly European *Sterreria* subgroup and blue the extra-European *Sterreria*; the distant *S. martindalei* n.sp. and *S. papuensis* n.sp. are shown in orange, as they was not tested with BP&P (s. text). Bootstrap support and Bayesian posterior probabilities are projected from different ML and Bayesian analyses in the order LSU, SSU and H3 where topologies were congruent. Clades supported in at least two of the three gene trees, present as separate networks by statistical parsimony, represented by at least three specimens, and validated by multi locus Bayesian analysis (except *S. martindalei* n.sp. and *S. papuensis* n.sp., see text), are formally described and named in this paper. Clades represented by two or less specimens were considered too poorly known for formal description but represent hypothetical species shown here with abbreviations (e.g. N1, S2).

**Figure 3:**
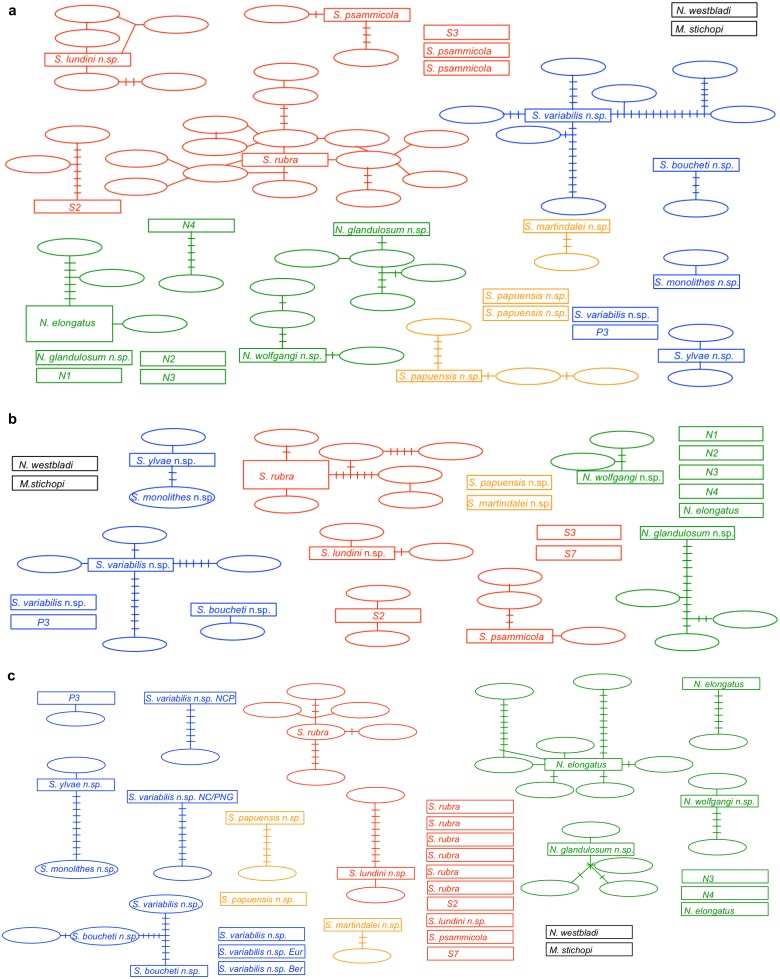
Parsimony haplotype networks calculated with TCS. a) LSU gene dataset, b) SSU gene dataset and c) Histone 3 gene dataset. The datasets were reduced and trimmed in order to reduce artefacts from missing data. The colours indicate the subgroups *Nemertinoides* (green), mainly European *Sterreria* (red) and extra-European *Sterreria* species (blue), and the not validated *S. martindalei* n.sp. and *S. papuensis* n.sp. in orange (see Fig 2). Some haplotypes are not connected to any other haplotype given the threshold and are represented by single boxes.

Those clades that consisted of at least three specimens, showed an averaged interspecific pairwise distance at least two times higher than the intraspecific averaged pairwise distance (relative threshold distance [34]), formed separate parsimony networks and were present in at least two of the three gene trees, were tested using a multilocus Bayesian approach with the program BP&P to generate secondary species hypotheses [18], [35], species validation sensu [36]. The program relies on a user-defined tree and only tests for the presence of nodes in the input-tree; the input of an incorrect guide tree will corrupt the results [32]. In order to create unambiguous input trees the dataset was divided into three subsets and the putative species *Sterreria martindalei* n.sp. and *Sterreria papuensis* n.sp. were excluded (different colours in Fig. 2, 3) because the gene trees could not resolve all deeper nodes with high support. Both excluded species, however, are highly supported in all species discovery methods, thus we think that further validation in these cases was not necessary. The subgroups within *Sterreria variabilis* n.sp. were not validated because of the unresolved topology (polytomies) of the group. Two analyses with the gamma priors set to G(1, 100) and G(1, 1000) for the population size θ and G(1, 100) and G(1, 1000) for the root age τ were conducted while the other divergence time parameters are assigned the Dirichlet prior ([18]: equation 2). An additional analysis with an older root age with the G of θ (1, 100) and the G of τ (1, 10000) was also conducted.

The species we describe are diagnosed based on unique differences in the nucleotide sequences following Jörger and Schrödl [4] in addition to morphological diagnostic characters, which are provided where available.

### Nomenclatural acts

The electronic edition of this article conforms to the requirements of the amended International Code of Zoological Nomenclature, and hence the new names contained herein are available under that Code from the electronic edition of this article. This published work and the nomenclatural acts it contains have been registered in ZooBank, the online registration system for the ICZN. The ZooBank LSIDs (Life Science Identifiers) can be resolved and the associated information viewed through any standard web browser by appending the LSID to the prefix “http://zoobank.org/”. The LSID for this publication is: urn:lsid:zoobank.org:pub:A306F670-B4B4-4376-A859-48A9735E1593. LSIDs for new species are given in table 2. The electronic edition of this work was published in a journal with an ISSN, and has been archived and is available from the following digital repositories: PubMed Central, LOCKSS and DiVA (http://www.diva-portal.org/smash/search.jsf).

## Results

When testing for random similarity between sequences, Aliscore highlighted 419 of 3583 aligned sites in the LSU dataset and 110 of 1792 sites in the SSU dataset. No random similarities were indicated in the H3 dataset. Consensus and best trees resulting from analyses of the original and Aliscore-filtered alignments had identical topologies with the exception of four specimens of *Sterreria rubra* that grouped with the specimen S7 in the Aliscore pruned LSU analysis. There were small differences in branch support when comparing original and filtered alignments (greatest difference 6% BS in the LSU dataset and 55% in the SSU dataset, but generally no or less than 10% BS difference). The gene trees shown as supplementary data are based on the original alignments (Figures S2–7).

A summary of the results in terms of putative species is given in figure 2, which shows a 75% majority rule consensus tree (MF75) of the LSU ML analysis performed with RAxML. Support (bootstrap for ML analyses, posterior probabilities for Bayesian analyses) for the 20 putative species (excluding outgroups) is shown to the right of the node (or in case of a few long branches over those) in the order LSU/SSU/H3 (for all gene trees see Figures S2–7). The colours refer to the subsets used for species validation (green: *Nemertinoides*-group, red: European *Sterreria*-group, blue: extra-European *Sterreria*-group, orange: untested *Sterreria* species).

In the uncorrected pairwise distances matrix several groups with at least twice the intraspecific distance to their sister group could be identified (Tables S2–4). The LSU and SSU gene datasets each had 19 distinct putative species groups (excluding outgroups), and in the Histone 3 gene dataset we found 28 such groups. The intraspecific distances never exceeded 0.8% and 0.5% respectively in the LSU and SSU data partitions, with the exception of N4 with 1.2% in the LSU dataset. In the LSU dataset the single specimen representing putative species S7 was excluded from the pairwise distance analysis because the sequence was too short. In the SSU dataset the species *Sterreria ylvae* n.sp. and *S. monolithes* n.sp. could not be distinguished from each other (averaged interspecific pairwise distance 0.2%). In the H3 partition intraspecific distances reached 9.9% in *Sterreria variabilis* n.sp. Of the 27 groups in the H3 data, 15 correspond to the same putative species as seen in the LSU and SSU gene datasets (Table 4). Eight groups in the H3 dataset did not correspond to putative species supported by the other two genes. This may be a saturation artefact (Figure S1).

**Table 4:** Summary of the results of the different species identification methods (per genetic marker) and the multi-locus species validation (BP&P).

Species/clade		Number of specimenssequenced per gene(LSU/SSU/H3)	Smallest interspecific vs. intraspecificpairwise distance (uncorrected)			Parsimony networks			Gene trees (ML/Bayesian)			BP&P
			LSU	SSU	H3	LSU	SSU	H3	LSU	SSU	H3	
N1		1/1/−	2.9/−	10.6/0	−	−	yes	−	100/1.0	37/0.61	−	−
N2		2/2/−	4.8/−	3.0/0	−	yes	yes	−	100/1.0	100/1.0	−	−
N3		2/2/2	7.8/0	3.8/0	5.8/0.3	yes	yes	yes	100/1.0	100/1.0	100/1.0	−
N4		2/2/2	2.9/1.2	2.4/0	8.7/0.2	yes	yes	yes	100/1.0	100/1.0	100/1.0	−
*N. elongatus*		21/20/15	2.9/0.6	2.4/0.1	5.8/0.8	yes	yes	yes*	100/1.0	100/1.0	91/1.0	1/1/1
*N. wolfgangi* n.sp.		9/7/8	5.1/0.2	1.4/0.3	4.9/1.0	yes	yes	yes	100/1.0	−^#^/−^#^	97/0.99	1/1/1
*N. glandulosum* n.sp.		13/13/12	1.7/0.4	1.4/0.5	4.9/0.5	yes*	yes	yes	87/0.99	100/1.0	65/0.81	1/1/1
*S. rubra*		44/45/17	1.0/0.2	1.2/0.2	7.9/0.9	yes	yes	yes*	94/1.0	78/0.98	74^#^/0.99^#^	1/0/0.93
S2		3/3/1	1.5/0.3	0.8/0	7.9/0	yes	yes	yes	100/1.0	91/1.0	70/0.97	1/0/0.93
S3		1/1/−	1.0/−	0.8/0.1	−	yes	yes	−	100/1.0	83/1.0	−	−
*S. lundini* n.sp.		13/12/4	4.2/0.1	1.7/0.1	−	yes	yes	yes*	98/0.99	−^#^/−^#^	99^#^/1.0^#^	1/0/0.93
*S. psammicola*		6/6/2	4.2/0.8	1.7/0.2	13.4/0	yes*	yes	yes	98/1.0	100/1.0	100/1.0	1/0/0.93
*S. papuensis n.sp.*		10/10/4	12.8/0.6	3.4/0.1	10.6/0.3	yes*	yes	yes*	100/1.0	100/1.0	99^#^/0.98^#^	−
S7		1/1/1	−	3.4/0	10.6/0	−	yes	yes	100/1.0	100/1.0	−	−
*S. martindalei* n.sp.		3/3/3	13.7/0.2	5.5/0	13/0.1	yes	yes	yes	100/1.0	100/1.0	100/1.0	−
*S. ylvae* n.sp.		4/4/4	5.4/0	0.2/0.1	9.4/0.1	yes	2	10	99/1.0	93/0.58	97/0.94	1/1/1
*S. monolithes* n.sp.		3/3/3	5.4/−	0.2/0	9.6/0	yes	2	10	100/1.0	89/0.97	100/1.0	1/1/1
*S. boucheti* n.sp.		7/7/6	6.4/0.1	5.2/0	14.2/1.4	yes	yes	5	100/1.0	100/1.0	46/0.65	1/1/1
P3		1/1/2	14.1/−	8.3/0	9.4/0.3	yes	yes	yes	100/1.0	98/1.0	100/1.0	−
*S. variabilis* n.sp.		16/16/14	6.4/0.6	5.2/0.3	14.8/9.9	yes*	yes*	no	95/0.99	80/0.78	7^#^/0.88	1/1/1
	NCP	2/2/2	−	−	12.5/2.1	−	−	yes	100/1.0	99/0.91	−/0.96	−
	NC/PNG/H	4/5/4	−	−	8.6/3.8	−	−	yes*	−/−	−/−	56/0.92	−
	Eur	5/5/4	−	−	8.6/0	−	−	yes	−/−	−/−	100/1.0	−
	Ber	4/4/4	−	−	13.1/0	−	−	yes	−/−	98/1.0	100/1.0	−

The number of specimens is given per gene in the order LSU, SSU and H3. The uncorrected percentage of pairwise distances was calculated on the same reduced dataset as was used for the calculation of the parsimony networks; the lowest average percentage of pairwise distance between any two clades within the datasets versus the averaged intraspecific pairwise distance is shown. The parsimony networks were calculated with a threshold of 98% for LSU and SSU and 90% for H3; numbers specify the steps between the two clades in case they were recovered in one network, an asterisk indicates that a given clade was recovered in more than one network. Gene trees were calculated using RAxML and MrBayes, support is given in this order; non-monophyletic clades are indicated with a hash in superscript (^#^). The program BP&P relies on a true guide tree, which due to the low taxon sampling we could not provide. We therefore decided to test only species with at least three specimens, thus some of the clades in the dataset were not tested and will not be named in this paper (except for S. martindalei and S. papuensis, which were well supported and described without BP&P validation). The posterior probabilities of the three different BP&P analyses are shown in the table. Due to the high variability of S. variabilis clade in all analyses, the clusters within this clade were tested for all analyses with the H3 gene. However, as this part of the tree was not resolved in either phylogenetic analysis, so no BP&B validation could be performed. A dash indicates missing data, or not tested clades.

The TCS software defined different numbers of parsimony haplotype networks for each of the three loci. Analyses of the LSU, SSU and H3 gene datasets with a connection limit of 98% found 25, 20 and 42 networks respectively (excluding outgroups, Fig. 3a, b). When the Histone 3 gene analysis was relaxed with a connection limit of 90% only 30 networks were found (Fig. 3c). In the LSU dataset *N. glandulosum* n.sp., *S. papuensis* n.sp., *S. psammicola* and *S. variabilis* were recovered as two and three separate networks respectively. Putative species S7 was excluded from the dataset due to its short sequence. In the SSU analysis, *S. boucheti* n.sp. and *S. ylvae* n.sp. were recovered as one network with two steps between the two species. One specimen of the diverse *S. variabilis* n.sp. formed a separate network not connected to the other specimens of the species. The H3 gene analyses split *S. lundini* n.sp. and *S. papuensis* n.sp. into two networks each and *N. elongatus* into three different networks. *S. rubra* was recovered in seven networks most of them consisting of only one or two specimens, corresponding with the observed pairwise distances. *S. variabilis* n.sp. formed five networks and one network connecting with *S. boucheti* n.sp. *S. ylvae* n.sp. and *S. monolithes* n.sp. formed one network connected by ten steps. In summary the network assemblages discovered with TCS are highly congruent with the groups identified in the pairwise distance matrix between genes, especially in the LSU and SSU genes. Tajima’s D for the H3 dataset is D = 1.931985, which indicates that this marker underwent neutral evolution. The H3 saturation test indicates saturation (S1).

The gene trees of the three loci estimated with RAxMl (best tree with bootstrap values) and MrBayes are not resolved in the deeper topology but consistently support the same putative species (Fig. 2, Figures S2–7). In the LSU and SSU genes 20 putative species (excluding outgroups) can be identified and in the Histone 3 dataset 27 such groups are supported. The groups identified in the gene trees are identical or highly congruent with the groups identified in the pairwise distance analyses and by the haplotype networks.

In the H3 gene trees thirteen of the 101 ingroup specimens are recovered as one clade splitting from a basal trichotomy. These specimens belong to *S. lundini* n.sp., *S. papuensis* n.sp. and *S. rubra*. This grouping can be interpreted as a saturation artefact. Exclusion of these specimens from the analyses did not change the composition of other tip groups (putative species). The recovered putative species in the gene trees, other than *S. lundini* n.sp. and *S. papuensis* n.sp., are consistent with those identified from the pairwise distances and haplotype networks.

Table 4 summarizes the support for identified groups over all methods of discovery.

### Species validation

**Figure 4:**
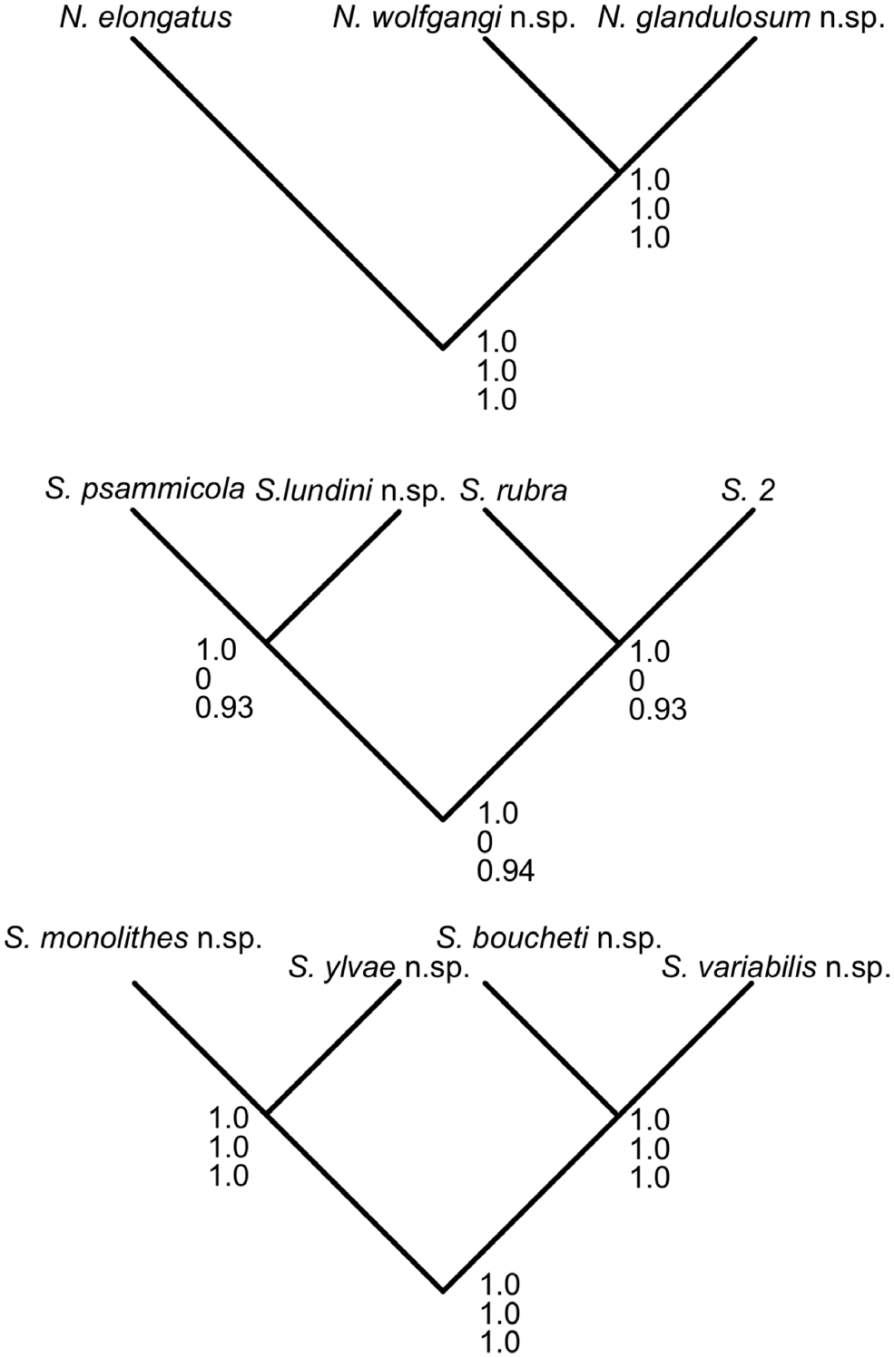
Results of the BP&P analyses for the tested species. Results given as nodal support for all *Nemertinoides* species (green in Fig 2), mainly European *Sterreria* species (red in Fig 2) and the extra-European *Sterreria* species (blue in Fig 2). Support values are Bayesian posterior probabilities for the different analyses in the order G(1/100), G(1/1000) and old root age (G(1/100) and G(1/10000)). The dataset was split in order to avoid artefacts due to unresolved topologies in the gene trees and increase confidence in the input topologies. Only clades represented by more than two specimens were tested in order to increase confidence.

The BP&P analyses with less informative priors or an older root age supported all eleven putative species tested (Fig. 4). The analysis with an informative prior supported all putative species except *Sterreria lundini* n.sp., *S. psammicola*, *S. rubra* and S2 (Fig. 4).

All 35 specimens with any kind of rosy, brownish to bright red colouration recorded were found in the species *Sterreria rubra* and clade S2 together with four uncoloured specimens and seven specimens for which no colour data was recorded.

**Figure 5:**
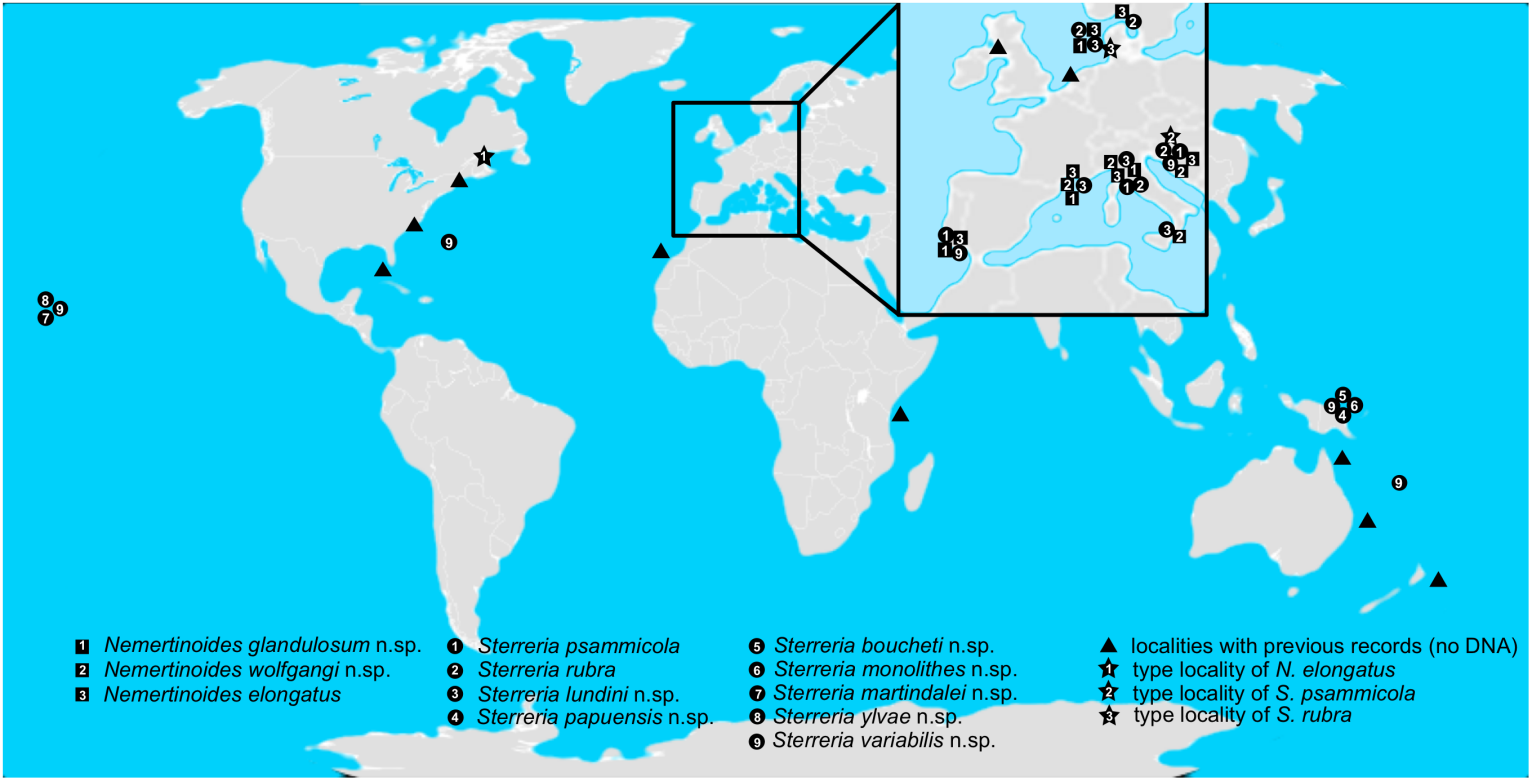
Distribution map. World map showing the distribution of all named species of Nemertodermatida in this study, Europe is shown in an expanded view. Records for presence of *Nemertinoides* species are shown as squares, *Sterreria* species as dots with numbers corresponding to the species (see legend within Figure). Localities with records for “filiform” Nemertodermatida from the literature are marked as triangles and type localities of the previously described species are shown as stars.

Seven out of the eight *Sterreria* species are not globally distributed, with two species being limited to Hawaii and Papua New Guinea respectively (Fig. 5). Only one species, *S. variabilis* n.sp., includes specimens from Hawaii, Papua New Guinea, New Caledonia, Bermuda, Portugal and the Mediterranean.

All three *Nemertinoides* species are distributed along the European coastline from the Mediterranean, via Portugal and the North Sea to the Swedish West Coast (Fig. 5). Within each clade however, no patterning corresponding to geographical distances could be observed. The same is true for the putative species indicated with red (Figs. 2 and 5), which also consist of European specimens and show no geographic pattern.

Clades meeting the above mentioned criteria and were supported in the BP&P analyses (except *S. martindalei* n.sp. and *S. variabilis* n.sp.) are formally described, with the exception of clade S2; the specimens of this clade lack photographs or sketches, prohibiting formal description of the species (Tab. 4).

## Discussion

Our dataset multiplies the available nucleotide sequence data for Nemertodermatida more than 100 times. The analyses of nucleotide sequence data from the three genes for LSU rRNA, SSU rRNA and Histone 3 identified twelve well supported species among the collected specimens from the two nominal species *Nemertinoides elongatus* and *Sterreria psammicola* (Tab. 4). *N. wolfgangi* n.sp. and *S. lundini* n.sp. are supported in all genes and analyses but are paraphyletic in the SSU gene trees. The unified species concept defines species as a “separately evolving lineage segment” [19]. SSU has been shown to underestimate species diversity in meiofauna [37]. We therefore conclude that both species are independently evolving lineages warranting formal description as species with incomplete lineage sorting in the SSU gene.

Twelve described species, however, clearly is an underestimation of the true biodiversity in this group of Nemertodermatida (Fig. 2). Even in the material from the Mediterranean, the most densely sampled geographical area, there are several clusters of less than three specimens consistently grouping together in all analyses. These clades represent additional cryptic diversity but we refrain from formally naming such poorly sampled putative species here. This taxonomic undersampling is of course even more drastic outside the Mediterranean. A different form of undersampling emanates from the limited dataset that we have acquired: inclusion of additional molecular markers would have boosted the potential to detect additional cryptic species. Our conservative approach, based on three molecular markers, still raises the number of named species of *Nemertinoides* and *Sterreria* from two to twelve.

With the material available to us, morphological distinguishing characters could not be identified *a priori* for all of the herein described species while studying live specimens, but it is possible that such features will be discovered *a posteriori* if more material becomes available. We suspect that this is a matter of the level of detail in the morphological investigation, which could be extended from light microscopy to CLSM or electron microscopy in search of additional characters. Even if some of the new species remain diagnosable only based on nucleotide sequences, it is important to recognize such cryptic species in order to appreciate biodiversity, plan management of conservation, and understand ecosystem function [14] (with references). This is especially true if much of species diversity is constituted by cryptic taxa, as is evidently the case in *Sterreria* and *Nemertinoides*. It has been argued that species delimitations based solely on nucleotide sequence data would lead to taxonomic instability and confusion as well as taxonomic inflation [38]. However, multilocus coalescent-based methods for species delimitation are firmly grounded in evolutionary theory and population biology, and since these methods are based on explicit probabilities they can be considered more objective than traditional character based taxonomy and allow greater comparability between species [39], [40]. Furthermore, if nucleotide-based species diagnoses were implemented, juvenile specimens as well as fragments of specimens, a large proportion of the nemertodermatid specimens encountered, would be available for the study of the diversity of this group.

Adams *et al.* 2014 [41] defined hyper-cryptic species as nominal species that actually consist of four or more valid species. Our application of molecular species discovery tools have revealed that the two nominal species *Nemertinoides elongatus* and *Sterreria psammicola* are hyper-cryptic as they are composed of at least 20 separate species-level clades. Nine of these will be formally described and named below, thereby doubling the number of nominal species of Nemertodermatida. There is no reason to believe that Nemertodermatida are unique in their extensive cryptic diversity: analyses of nucleotide sequence data have unravelled cryptic and hyper-cryptic species within many other groups of marine invertebrates. A case in point is the nominal polychaete species *Eumida sanguinea* (Örsted, 1843) which was studied by Nygren and Pleijel [42] who identified eight cryptic species among specimens assigned to *E. sanguinea* and named seven of them using nucleotide-based diagnoses. There are a number of additional cases of cryptic diversity in other polychaete taxa, e.g. the “cosmopolitan” fireworm *Eurythoe complanata* (Pallas 1766), which was found to consist of three species [43], and *Notophyllum foliosum* (Sars 1835), which was found to consist of two species [44]. There are relatively few studies at this level of taxonomic resolution in marine meiofauna, but cryptic species have been identified in the flatworm genera *Pseudomonocelis* [45], [46] and *Monocelis* [47]. A noteworthy example is the “cosmopolitan” flatworm *Gyratrix hermaphroditus* Ehrenberg, 1831, where studies of karyotype and fine morphology revealed eight separate species in Australia [48], two separate species in the North Sea and the Mediterranean and two separate species at the French Atlantic coast [49]. Leasi *et al.* [50] used the coalescent-based GMYC algorithm [51] to analyse Cytochrome oxidase subunit I sequences from specimens of the rotifer *Testudinella clypeata* (Müller, 1786) and found seven cryptic species. Our results further corroborate the hypothesis of the oceans as a hotspot for cryptic diversity put forward by Bickford *et al.* [14] and exemplified above. Cryptic species occur in all animal groups and they are being identified and described at an accelerating rate [14], [52], [53]. We hypothesize that the amount of cryptic diversity in meiofauna is far higher than the 11%–43% estimate proposed by Appeltans *et al.* [13]. Consequently, the approximation of total marine diversity at 0.7–1.0 million species is likely to be an underestimate.

As noted above, identification and taxonomic study of Nemertodermatida requires specialized methods and access to live specimens, which may be part of the explanation for our fragmentary knowledge of their diversity. This is true also for other groups of meiofauna, especially fragile groups such as Acoela, Platyhelminthes and Gastrotricha that cannot be easily preserved for future identification. Application of metagenetic methods e.g. [16], [54], [55], where DNA is extracted from sediment samples followed by PCR amplification of a selected marker, pyrosequencing and bioinformatic analysis, has potential to change this as the morphological identification stage is eliminated. This procedure is considered cost-effective in comparison to traditional methods where a number of taxonomists would have to study each sample [56]. It will also provide a more complete snapshot of diversity, as juvenile or damaged specimens would contribute to the results. Identification of species through the metagenetic approach requires a populated database of reference sequences from specimens that were identified by a specialist, such as those provided in this study.

### Habitat and Biogeography

All our specimens were found in depths between 1.5 m and 37 m in sand that reached from coarse to very fine with low to moderate organic content. However, we did not observe any differences in habitat specific to the identified species; if present they are clearly subtle. The nemertodermatid species *Nemertoderma westbladi* and *Meara stichopi*, outgroup species in this study, occur in mud down to depths of 600 m. Sampling of nemertodermatids from deeper sediments has only been done in very few locations, mainly in the North-East Atlantic. Thus the existence of deep-water species of *Sterreria* and *Nemertinoides* cannot be ruled out.

No fossils identified as nemertodermatids are known, but according to both of the two currently competing hypotheses on the phylogenetic position of Nemertodermatida [57], [58], the group is as old as the Cambrian explosion, or even predating it. When trying to explain the current distribution of such an old clade as the Nemertodermatida, and taking into account their biology with the poor capacity for dispersal that it implies, a first hypothesis may be to invoke vicariance, explaining the patterns by continental drift in combination with speciation. However, the vicariance hypothesis cannot explain the presence of littoral Nemertodermatida on younger and isolated islands such as Hawaii and Bermuda. O’ahu island, where our Hawaiian specimens were collected, is three million years old [59]. It should also be noted that the high estimated age of Nemertodermatida, deduced from their phylogenetic position, pertains to the clade as a whole. The age of the nemertodermatid crown group, which includes the recent species of *Sterreria* and *Nemertinoides,* cannot be determined in the absence of any calibration points, but it is likely to be much younger. Clearly, dispersal is the only feasible explanation for the presence of *Sterreria* on O’ahu and Bermuda. The possibility remains that isolated young islands were colonized by deepwater nemertodermatid species, although currently available evidence seems to favour dispersal from distant shallow habitats, as no deep-water *Sterreria* specimens are known. The phylogenetic hypothesis derived from Bayesian analyses of the ribosomal datasets indicates that O’ahu was colonized at least twice as *Sterreria ylvae* n.sp., *Sterreria martindalei* n.sp. and *Sterreria variabilis* n.sp. are not each others closest relatives. Current sampling of *Sterreria* on O’ahu was restricted to one site. Extended sampling of nemertodermatids in the Hawaiian archipelago, where the islands are of different ages ranging from 28 Myears to 400 000 years [59], would allow an estimate of rates of colonization and speciation within *Sterreria* as well as indicating the relative importance of dispersal and speciation in nemertodermatid diversity. Similar studies in Macaronesia, with its volcanic islands of different ages and degrees of geographical isolation, would also shed light on the genetic distinctness of the Bermudian species and the transatlantic dispersal.

Our results show that Nemertodermatida mostly do not conform with the EiE hypothesis. The supposedly wide-ranging *Sterreria psammicola* and *Nemertinoides elongatus* both consist of complexes of cryptic species. Some of the species, e.g. *Nemertinoides wolfgangi* n.sp., show a distribution pattern restricted to one ocean, in this case the Mediterranean, which is, as noted above, the best represented area in this study. However, other species have more extensive distribution areas, such as *Nemertinoides elongatus* and *Sterreria rubra*, ranging from the Adriatic, through the Mediterranean via Portugal and the North Sea into the Skagerrak without exhibiting any apparent genetic structure. This indicates considerable dispersal ability in these interstitial worms. Outside Europe, the findings in the Madang lagoon, Papua New Guinea, are particularly striking: the 25 animals collected from four adjacent localities (less than 10 km distance) belong to six different species (*S. papuensis* n.sp., *S. variabilis* n.sp., *S. boucheti* n.sp., *S. monolithes* n.sp., S7 and P3). Of those, *S. papuensis* n.sp. and S7 are more closely related to species with European distribution, than to species from geographically closer localities. This is clearly not consistent with an isolation by distance pattern and again indicates dispersal.

Only one species in our dataset appears to be truly cosmopolitan: *Sterreria variabilis* n.sp. However, since the Histone 3 gene splits this nominal species into geographically structured clades, the existence of yet another unresolved species complex is possible.

### Taxonomic part

#### Family: Nemertodermatidae Steinböck, 1930

Nemertodermatida without a female pore or bursa. Male pore supraterminal or dorsal. Sequential hermaphrodites. Sperm radially symmetric. Lithocyte in blisters. Usually with epidermal bottle glands.

#### Genus: *Nemertinoides* Riser, 1987

Diagnosis (emended): Nemertodermatidae with elongated body and constriction at level of statocyst. Mouth in anterior half of body, male pore variable at U40 or subterminal, testes post-oral, ovaries in posterior half of body, reaching posterior of male opening.

Remarks: The far anterior position of the male pore in *N. elongatus* with posterior ovaries were the main arguments for the naming of a new genus by Riser [11]. However, the position of the male pore in relation to body length proved to be not informative on genus level, as opposed to the relative position of the ovaries reaching posterior of the male opening.

### Three species

#### -*N. elongatus* Riser, 1987

Material examined: 21 living specimens in squeeze preparation collected during summer between the years 2007 and 2013 in western Sweden (6), the North Sea (1), southern Portugal (3), the French Mediterranean (1), the Tyrrhenian Sea (7), and the Adriatic (3). More detailed information about individuals and further photographs are accessible at http://acoela.myspecies.info/, the scratchpads database for Acoela and Nemertodermatida.

**Figure 6:**
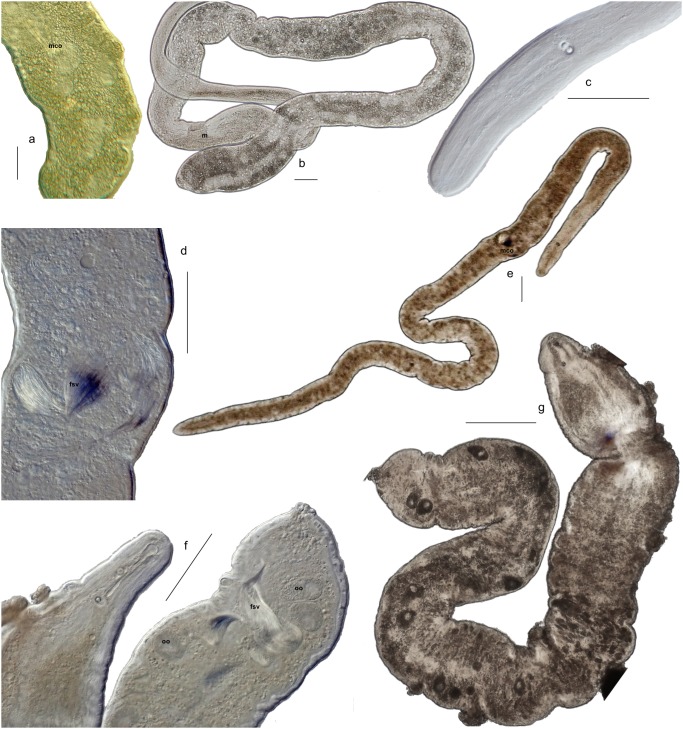
Diversity within the genus *Nemertinoides*. a–c: *Nemertinoides glandulosum* n.sp. a) Posterior with mco. b) Overview (anterior region missing) of worm with mouth (m). c) Anterior with statocyst with double statoliths and frontal organ. d, e: *Nemertinoides elongatus*. d) Detail of the male copulatory organ with false seminal vesicle. e) Overview of whole animal with position of the mco. f, g: *Nemertinoides wolfgangi* n.sp. f) Anterior and posterior of fully mature animal with oocytes and fsv in the posterior. Only one statoliths in statocyst visible as photo is taken slightly laterally. g) Overview over the same animal with oocutes still visible but no fsv. Photographs were taken of live specimens, b–g are photographs of the respective holotypes. The scale bars indicate 100 µm for each photograph.

Description: Up to 6 mm long, region anterior of statocyst slimmer than rest of body. Statocyst at U4. Large bottle glands in epidermis in anterior half of body. Mouth at U25. Male opening at U40 (Fig. 6e); false seminal vesicle (fsv) directly anterior to this (Fig. 6d). Paired ovaries extend from posterior of fsv to posterior tip. Found in slightly coarser sand from the intertidal to 30 m depth.

Diagnosis (emended): Molecular character diagnosis in table 5. Smallest interspecific pairwise distance vs. intraspecific distance 2.9/0.6% (LSU), 2.4/0.1% (SSU) and 5.8/0.8% (H3).

**Table 5:** Molecular Diagnostic character of all newly described species in the three genes used in this study.

	LSU	SSU	H3
*N. glandulosum* n.sp. *(13185)*	2493 (1323) T, 2506 (1336) A	175 (149) C, 696 (660) A, 725 (688) C,727 (690) C	43 (31) C, 70 (58) T, 79 (67) T, 103 (91) T
*N. wolfgangi* n.sp. *(13453)*	2450 (1292) T, 2493 (1333) C,2603 (1443) C	192 (135) G	79 G, 103 C, 262 T
*S. boucheti* n.sp. *(PNG75)*	2195 (1060) G, 2205 (1070)G, 2333 (1198) G,2487 (1288) A, 2510 (1331) A	631 (579) G, 696 (643) A, 723 (669) A,827 (770) A	−
*S. lundini* n.sp. *(08117)*	1705–1708 (1463–1467) CTCTC (insert)	701 (632) T, 780 (705) C	−
*S. martindalei* n.sp. *(10056)*	2037 (1611) A, 2048 (1622) C,2123 (1690) C,2193 (1757) G, 2466 (2021) A,2523 (2061) G	95 (91) A, 115 (110) G, 127 (122) C,257 (235) T, 263 (241) T, 530 (506) T,594 (570) C	26 (11) A, 28 (13) A
*S. monolithes* n.sp. *(PNG84)*	2003 (1642) T, 2326 (1953) G,2506 (2102) T,2518 (2113) C, 2604 (2199) A	204 (182) C, 205 (183) A, 682 (651) A	100 (88) A, 223 (211) T, 124 (112) G
*S. papuensis* n.sp. *(PNG48)*	1825 (718) T, 1848 (727) A,1860 (735) C, 2099(963) A, 2337 (1192) T, 2453 (1290) T	698 (671) T, 711 (683) G, 780 (746) T	40 (9) G, 82 (51) C, 118 (87) A
*S. psammicola (13508)*	1696 (1105) T, 1699 (1108) G,1704 (1113) G, 1714 (1119) A	701 (101) A, 710 (110) A, 775 (172) C	61 G, 67 T, 73 A, 136 A, 142 A, 151 G
*S. variabilis* n.sp. *(13452)*	2343 (1208) C, 2419 (1261) A,2995 (1804) A	631 (502) T, 681 (551) C, 723 (592) G,827 (693) C	−
*S. ylvae* n.sp. *(10054)*	2003 (1642) C, 2431 (2034) T,2490 (2086) A, 2601 (2195) A	204 (183) T, 1717 (1658) A	124 (112) A

Numbers refer to positions in the respective alignments and in brackets to the position in the sequences in the type specimen (genbank accession number).

Remarks: This species conforms to the description of *N. elongatus* in Riser [11] with the position of the male opening at about U40 and the distribution of epidermal glands only in the anterior half of the body length. Species supported by all three genes in this study. The type material for *N. elongatus* was collected in the Western Atlantic (holotype at the Massachusetts coast and paratypes at the New Brunswick coast). Subsequent specimens identified as *N. elongatus* were collected in the Adriatic by Sterrer [9]. There is some uncertainty attached to the identification of this species, as we did not have access to specimens from the type localities; it is possible that our specimens represent a species different from the original *N. elongatus.*

Distribution: Western Atlantic, Swedish West coast, North Sea, southern Portugal, Mediterranean.

#### -*Nemertinoides wolfgangi* n.sp

Material examined: 9 living specimens in squeeze preparation collected mostly in summer between the years 2008 and 2013 in the French Mediterranean (1), the Tyrrhenian Sea (5), Sicily (2), and the Adriatic (1). More detailed information about individuals and further photographs are accessible at http://acoela.myspecies.info/, the scratchpads database for Acoela and Nemertodermatida.

Typematerial: Holotype SMNH type-8632 (collection code UJ-13453). Mature specimen collected near Cherso, Croatia, by Marco Curini Galletti 22 September 2013. Photographs of the holotype specimen deposited at the Swedish Museum of Natural History, Stockholm.

Description: Reaching more than 5 mm in length. Anterior narrow with body comparatively wide, wobbly. Posterior rounded (Fig. 6f, g). Male copulatory organ (mco) at U90 (Fig. 6f). Paired ovaries in posterior half of body, also posterior of mco (Fig. 6e). Statocyst anterior of U10. Frontal glands open centrally at anterior tip, reaching to about U30. Epidermal bottle glands abundant all over body.

Diagnosis: Morphologically not clearly distinguishable from *N. glandulosum* n.sp. Molecular character diagnosis in table 5. Smallest interspecific pairwise distance vs. intraspecific distance 2.0/0.2% (LSU), 1.4/0.3% (SSU) and 4.9/1.0% (H3).

Remarks: Species supported by all three genes in this study, but is paraphyletic in the SSU gene tree.

Distribution: Mediterranean.

Etymology: After Wolfgang Sterrer, who published material collected over 35 years and reignited interest in the taxon and hosted I. M-W during the collection on Bermuda.

#### -*Nemertinoides glandulosum* n.sp

Material examined: 13 living specimens in squeeze preparation collected mostly in summer between the years 2007 and 2013 in the North Sea (2), southern Portugal (2), the French Mediterranean (3), and the Tyrrhenian Sea (6). More detailed information about individuals and further photographs are accessible at http://acoela.myspecies.info/, the scratchpads database for Acoela and Nemertodermatida.

Typematerial: Holotype SMNH type-8631 (collection code UJ-13185), mature specimen collected near Faro, Portugal, by Inga Meyer-Wachsmuth 23 May 2013. Photographs of the holotype deposited at the Swedish Museum of Natural History, Stockholm.

Description: Up to 5 mm long. Anterior part narrow compared to wider, wobblier body. Epidermis thick and glandular (Fig. 6b). Posterior more pointy (Fig. 6b). Mouth at U35 (Fig. 6b). Statocyst anterior of U10. Frontal glands open centrally, secretions globular and connected by fine thread like pearls on a chain (Fig. 6c). Mco far in posterior (Fig. 6a).

Diagnosis: Although generally more slender and with slightly more pointy posterior, morphologically not clearly distinguishable from *N. wolfgangi* n.sp. Molecular character diagnosis in table 5. Smallest interspecific pairwise distance vs. intraspecific distance 2.0/0.4% (LSU), 1.4/0.5% (SSU) and 4.9/0.5% (H3).

Remarks: Species supported by all three genes in this study.

Distribution: Mediterranean, southern Portugal, North Sea.

Etymology: Glandula  =  latin for gland. This species, in contrast to the prior described *N. elongatus*, has epidermal glands also in posterior.

#### Genus: *Sterreria* Lundin, 2000

Diagnosis (emended): Elongated Nemertodermatidae without head constriction. Statocyst more posterior than in *Nemertinoides*. Mouth ventral at U50. Paired testes follicular. Male pore opens dorsally at U90, fsv anterior to that. Paired ovaries anterior of mco; oocytes mature caudad.

Remarks: The species *Sterreria psammicola* has been described originally within the genus *Nemertoderma* by Sterrer in Riedl (1970). Due to differences in epidermal structure and differences in the position of the reproductive glands Lundin [60] created a new monotypic genus *Sterreria* and placed *Sterreria psammicola* in this. The mouth has been observed only in few specimens, it is hypothesized to be a temporary feature as in *Nemertoderma westbladi* [61]–[63]. “Male maturity seems to precede female maturity” [9].

### Nine species

#### -*Sterreria psammicola* (Sterrer, 1970)

Material examined: 6 living specimens in squeeze preparation collected mostly in summer between the years 2007 and 2013 in southern Portugal (2), the Tyrrhenian Sea (2), and the Adriatic (2). More detailed information about individuals and further photographs are accessible at http://acoela.myspecies.info/, the scratchpads database for Acoela and Nemertodermatida.

Typematerial: Neotype SMNH type-8640 (collection code UJ-13508). Mature specimen collected by Marco Curini Galletti 28 September 2013 at Miramare near Trieste, Italy.

**Figure 7:**
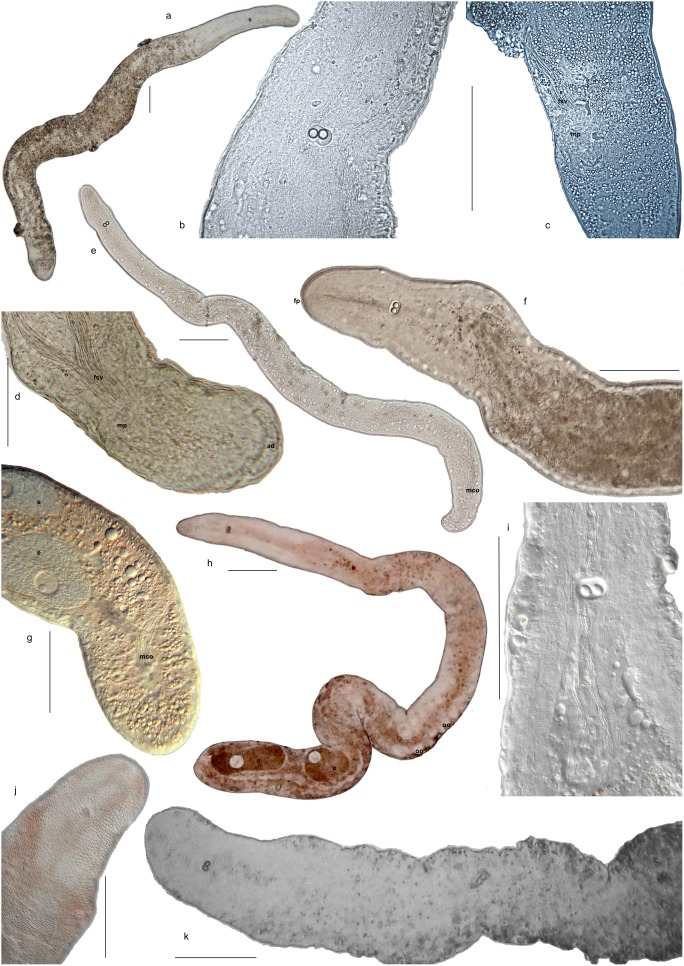
Diversity within the genus *Sterreria.* a–c: *Sterreria lundini* n.sp. a) Overview over whole animal. b) Anterior with statocyst with double statoliths and oval frontal gland secretions. c) Posterior with fsv and male pore (mp). d, e: *Sterreria psammicola*. d) Posterior with fsv male pore (mp) and adhesive area (ad). e) Overview over male mature animal with large epidermal glands distributed more or less evenly over the whole body and mco in the posterior. f) Anterior part of an animal with epidermal glands, statocyst and central frontal gland opening (fp). g–j: *Sterreria rubra*. g) Posterior with two mature eggs (e) and mco. h) Overview over the same male and female mature specimen with mature eggs (e) and oocytes (oo). i) Anterior with statocyst and rod-shaped frontal gland secretions. j) Detail of the epidermis in the anterior of an animal with cell borders clearly visible, giving the animal a “scaly” appearance. k: *Stererria papuensis* n.sp. Anterior part of the holotype. All photographs are taken of live specimens in squeeze preparation. a, b, c, f and k are photographs of the holotype and neotype specimens respectively. The scale bars indicate 100 µm for each photograph.

Description: Colourless (Fig. 7e). Frontal glands prominent, opening centred at anterior tip, secretions small, globular or oval (Fig. 7f). Epidermal glands distributed equally over body (Fig. 7e). Borders between epidermal cells not clearly visible (no “scaly” appearance). Posterior rounded, with adhesive area (Fig. 7d). Statocyst further constricted between statoliths than in other groups.

Diagnosis (emended): Morphologically not clearly distinguishable from *S. lundini* n.sp. Molecular character diagnosis in table 5. Smallest interspecific pairwise distance vs. intraspecific distance 4.2/0.8% (LSU), 1.7/0.2% (SSU) and 13.4/0% (H3).

Remarks: *Sterreria psammicola* has been described from Croatia as a filiform worm living in shallow sandy sediments under the name *Nemertoderma psammicola* by Sterrer in Riedl [64]. The species was described based on some specimens in which “a salmon-red longitudinal stripe is *usually* (italics by the present authors) present in the first third of the body length” [64]. Specimens from this clade conform to the description without colouration and two specimens were collected near the type locality of *Sterreria psammicola* in Croatia. In the formal description in 1970 no type material was deposited. Species supported by all three genes in this study.

Distribution: Mediterranean, southern Portugal.

#### -*Sterreria rubra* (Faubel, 1976)

Material examined: 45 living specimens in squeeze preparation collected mostly in summer between 2007 and 2013 in western Sweden (1), the North Sea (6), southern Portugal (6), the Tyrrhenian Sea (29), and the Adriatic (3). More detailed information about individuals and further photographs are accessible at http://acoela.myspecies.info/, the scratchpads database for Acoela and Nemertodermatida.

Description: Usually pigmented, rose, bright red or brownish; only in anterior part or all over body (Fig. 1a, b; 7 g–j). Frontal glands prominent reaching far behind statocyst; opening fanning out at the anterior tip; secretions rod shaped (Fig. 7i). Epidermal glands small, distributed densely especially in anterior third of body, but never as prominent as in *Nemertinoides*-species. Body surface appears “scaly” due to visible epidermal cell borders (Fig. 7j). Testes lateral. Male pore at U90; fsv just anterior to that (Fig. 7g). Ovaries paired, usually two mature eggs and several small oocytes (Fig. 7h). Posterior tip wide.

Diagnosis (emended): Usually pigmented. Secretions of frontal gland rod-shaped. Molecular character diagnosis in table 5. Smallest interspecific pairwise distance vs. intraspecific distance 1.0/0.2% (LSU), 1.2/0.2% (SSU) and 7.9/0.9% (H3).

Remarks: In 1976 Faubel described the species *Nemertoderma rubra* from the islands Rømø and Sylt in the North Sea based on three specimens and stated that “in transmitted light the species is coloured reddish” [10]. In a revision of the taxon Nemertodermatida, Sterrer [9] remarked that *Nemertoderma rubra* and *N. psammicola* are very similar and regarded them as one species, making *N. rubra* a junior synonym of *N. psammicola*. Due to the specimens of this clade conforming to the comprehensive formal description of *Nemertoderma rubra* and the clear statement that “the species is coloured reddish” we reinstate the junior synonym *Sterreria* (*Nemertoderma*) *rubra*. Ovaries are positioned further towards the posterior than described by Sterrer [9] and Faubel [10]. Species supported by all three genes in this study, but polyphyletic in the H3 gene tree (saturation artefact).

Distribution: North Sea, Swedish West coast, southern Portugal, Mediterranean.

#### -*Sterreria lundini* n.sp

Material examined: 12 living specimens in squeeze preparation collected mostly in summer between the years 2008 and 2013 in the North Sea (1), the French Mediterranean (1), the Tyrrhenian Sea (9), and Sicily (1). More detailed information about individuals and further photographs are accessible at http://acoela.myspecies.info/, the scratchpads database for Acoela and Nemertodermatida.

Typematerial: Holotype SMNH type-8634 (collection code UJ-08117). Male mature specimen collected near Castiglione della Pescaia, Italy, by Marco Curini Galletti 19 May 2008. Photographs of the holotype deposited at the Swedish Museum of Natural History, Stockholm.

Description: Up to 4 mm long. Colourless (Fig. 7a). Epidermal glands distributed over whole body. Frontal glands prominent with globular or oval secretions (Fig. 7b). Male pore at U90 (Fig. 7c). Posterior end rounded. Borders between epidermal cells not clearly visible.

Diagnosis: Morphologically not clearly distinguishable from *S. psammicola*. Molecular character diagnosis in table 5. Smallest interspecific pairwise distance vs. intraspecific distance 1.4/0.1% (LSU) and 1.7/0.1% (SSU).

Remarks: This species is paraphyletic in the SSU gene tree and polyphyletic in the H3 gene tree (saturation artefact).

Distribution: Mediterranean.

Etymology: After Kennet Lundin, the first researcher formulating a comprehensive phylogenetic hypothesis for Nemertodermatida.

#### -*Sterreria papuensis* n.sp

Material examined: 10 living specimens in squeeze preparation collected in November 2012 near four different islands (Siar, Tab, Panab, Wanad) in Madang Lagoon, Papua New Guinea. More detailed information about individuals and further photographs are accessible at http://acoela.myspecies.info/, the scratchpads database for Acoela and Nemertodermatida.

Typematerial: Holotype SMNH type-8637 (collection code PNG77). Immature specimen collected near Wanad island in November 2012 by Inga Meyer-Wachsmuth. Photos of the holotype deposited at the Swedish Museum of Natural History, Stockholm.

Description: Up to 1 cm long. Opaque, dirty rose. Frontal glands reach far towards posterior branching tree-like, opening fanning out. Mouth at U35. Male opening at U85. Adhesive structure in posterior. Borders between epidermal cells visible (“scaly”) similar to *Sterreria rubra*. Epidermal glands few but regularly distributed.

Diagnosis: Rosy, never bright red. Molecular character diagnosis in table 5. Smallest interspecific pairwise distance vs. intraspecific distance 12.6/0.6% (LSU), 3.4/0.1% (SSU) and 10.6/0.3% (H3).

Remarks: Species supported by all genes in this study but is polyphyletic in the H3 gene tree (saturation artefact).

Distribution: Madang Lagoon, Papua New Guinea.

Etymology: papuensis = coming from Papua (New Guinea). This is remarkable as this species is closely related to otherwise exclusively European (putative) species.

#### -*Sterreria boucheti* n.sp

Material examined: 7 living specimens in squeeze preparation collected in November 2012 near two different islands (Panab, Wanad) in Madang Lagoon, Papua New Guinea. More detailed information about individuals and further photographs are accessible at http://acoela.myspecies.info/, the scratchpads database for Acoela and Nemertodermatida.

Typematerial: Holotype SMNH type-8633 (collection code PNG75). One immature specimen collected near Wanad Island, Papua New Guinea, by Inga Meyer-Wachsmuth in November 2012. Photographs of the holotype deposited at the Swedish Museum of Natural History, Stockholm.

Description: 1.5 mm long. Colourless. Frontal glands open centrally, secretions rod-shaped, reaching posterior until U35. Large epidermal glands all over body. Statoliths in statocyst well separated. Follicular testes interspersed with ovary. Male pore at U90; mature sperm converge to fsv anterior of that.

Diagnosis: This species is delineated molecularly by all three genes used in this study. Molecular character diagnosis in table 5. Smallest interspecific pairwise distance vs. intraspecific distance 4.8/0.1% (LSU), 5.2/0% (SSU) and 14.2/1.4% (H3).

Remarks: Species supported by all three genes in this study.

Distribution: Madang Lagoon, Papua New Guinea.

Etymology: After Professor Philippe Bouchet, who hosted I. M-W at the 2012 Biodiversity expedition (www.ourplanetreviewed.org) and thus made safe sampling in a place as remote as Papua New Guinea possible.

#### -*Sterreria monolithes* n.sp

Material examined: 3 living specimens in squeeze preparation collected in November 2012 near two different islands (Panab, Wanad) in Madang Lagoon, Papua New Guinea. More detailed information about individuals and further photographs are accessible at http://acoela.myspecies.info/, the scratchpads database for Acoela and Nemertodermatida.

Type material: Holotype SMNH type-8636 (collection code PNG 84). Immature specimen collected near Wanad Island, Papua New Guinea, by Inga Meyer-Wachsmuth in November 2012. Photographs of the holotype deposited at the Swedish Museum of Natural History, Stockholm.

Description: Opaque silvery glossy, no pigmentation. Frontal glands open fanning out; rod-shaped criss-crossing secretions.

Diagnosis: This species cannot be morphologically delineated. It is supported by all three genes used in this study, molecular character diagnosis in table 5. Smallest interspecific pairwise distance vs. intraspecific distance 2.6/0% (LSU), 0.1/0% (SSU) and 9.6/0% (H3).

Remarks: All three specimens of species had only one statolith in the statocyst. Aberrant numbers of statoliths can be observed frequently in other species of Nemertodermatida without taxonomic implications. However, it is unusual to find only specimens with an abnormal number of statoliths. All studied specimens were immature.

Distribution: Madang lagoon, Papua New Guinea.

Etymology: monos = greek for single, lithos = greek for stone, referring to the single lithocyte in the statocyst in all three studied specimens.

#### -*Sterreria ylvae* n.sp

Examined material: 4 living specimens in squeeze preparation collected in May 2010 at Waimanolo beach, O’ahu island, Hawaii by Ulf and Ylva Jondelius. More detailed information about individuals and further photographs are accessible at http://acoela.myspecies.info/, the scratchpads database for Acoela and Nemertodermatida.

Type: Holotype SMNH type-8639 (collection code UJ-10054). One mature specimen collected at Waimanolo, Hawaii, by Ulf Jondelius 31. May 2010. Photographs of the holotype deposited at the Swedish Museum of Natural History, Stockholm.

**Figure 8:**
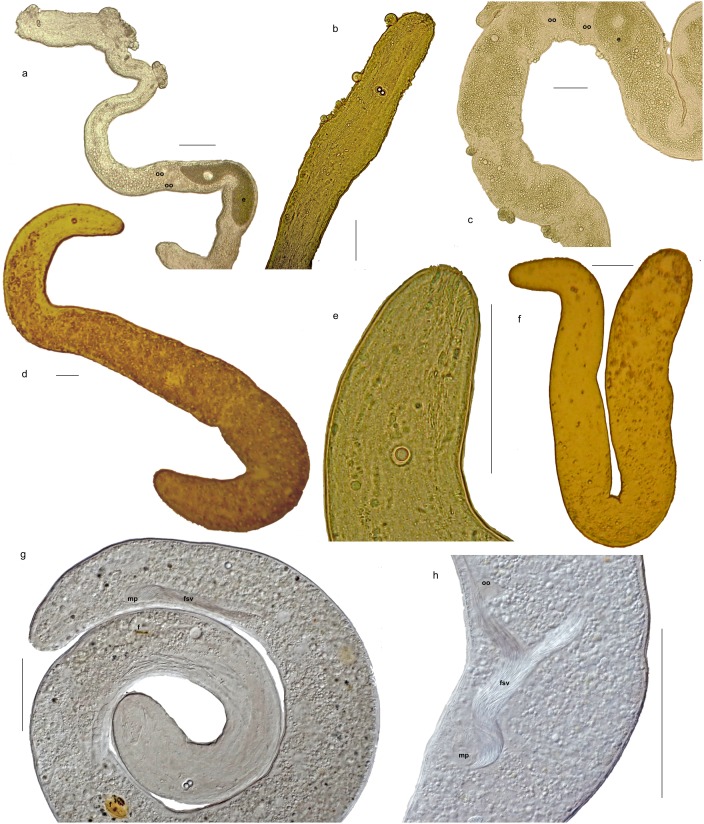
Diversity within the genus *Sterreria*. a: *Sterreria martindalei* n.sp. Overview over slightly damaged specimen with mature egg (e) and oocytes in the posterior. b, c: *Sterreria ylvae* n.sp. b) Anterior part with statocyst with double statoliths. c) Mid-section of the body with one mature egg (e) and few oocytes (oo). d, e: *Sterreria monolithes* n.sp. d) Overview over immature animal with only one statolith in the statocyst. e) Detail of the anterior of the same specimen with fanning out frontal gland opening and frontal gland secretions. The single statolith fills nearly the whole statocyst. f: *Sterreria boucheti* n.sp. Overview over complete, immature specimen. g, h: *Sterreria variabilis* n.sp. g) Overview over male mature specimens with club-shaped frontal gland secretions and food particles (f) in gut. h) Detail of male copulatory organ with fsv anterior of male pore (mp) and oocyte (oo). All photographs were taken of the live holotype specimens in squeeze preparation. The scale bars indicate 100 µm for each photograph.

Description: Statocyst positioned at U15, statoliths appear encapsulated into separate membrane inside statocyst (Fig. 8b). Anterior long and slender, body slightly wider, wobbly. Posterior narrow and rounded. Epidermal glands large, prominent in anterior half of body. Uncoloured, transparent, opaque only in mid-section of body (Fig. 8c).

Diagnosis: Statoliths appear to be encapsulated into separate membrane inside statocyst. Molecular character diagnosis in table 5. Smallest interspecific pairwise distance vs. intraspecific distance 2.6/0% (LSU), 0.2/0.1% (SSU), and 9.4/0.1% (H3).

Remarks: Delineated by all three genes used in this study.

Distribution: O‘ahu Island, Hawaii, USA.

Etymology: Named after Ylva Jondelius, who collected the samples at Waimanolo beach.

#### -*Sterreria martindalei* n.sp

Examined material: 3 living specimens in squeeze preparation collected in May 2010 at Waimanolo beach, O’ahu island, Hawaii by Ulf Jondelius. More detailed information about individuals and further photographs are accessible at http://acoela.myspecies.info/, the scratchpads database for Acoela and Nemertodermatida.

Type: Holotype SMNH type-8635 (collection code UJ-10056). One mature specimen collected at Waimanolo, Hawaii, by Ulf Jondelius 31. May 2010. Photographs of the holotype deposited at the Swedish Museum of Natural History, Stockholm.

Description: Statocyst at U5. Overall larger, sturdier than *Sterreria ylvae* n.sp. Posterior two thirds opaque due to large and dense epidermal glands dorsally or ventrally, not laterally. Frontal gland opening central, secretions ellipsoid, reaching posterior till U35; clear border between frontal and epidermal glands. Ovaries between U50 and U75 (Fig. 8a).

Diagnosis: Statocyst more anterior than in *Sterreria ylvae* n.sp. and overall sturdier, however morphologically not clearly distinguishable from *Sterreriae ylvae* n.sp. Molecular character diagnosis in table 5. Smallest interspecific pairwise distance vs. intraspecific distance 12.0/0.2% (LSU), 5.5/0% (SSU) and 13.0/0.1% (H3).

Remarks: Species supported by all three genes in this study.

Distribution: O‘ahu Island, Hawaii, USA.

Etymology: After Prof. Mark Martindale, who hosted U. J. in Hawaii and made sampling possible.

#### -*Sterreria variabilis* n.sp

Material examined: 15 living specimens in squeeze preparation collected mainly in summer between the years 2008 and 2013 in the Adriatic (2), the Tyrrhenian Sea (2), southern Portugal (1), Bermuda (4), New Caledonia (4), Papua New Guinea (2), and Hawaii (1). More detailed information about individuals and further photographs are accessible at http://acoela.myspecies.info/, the scratchpads database for Acoela and Nemertodermatida.

Typematerial: Holotype SMNH type-8638 (collection code UJ-13452). One male mature specimen collected at Cherso, Croatia, collected by Marco Curini Galletti 22 September 2013. Photographs of the holotype deposited at the Swedish Museum of Natural History, Stockholm.

Description: Morphologically diverse. Bermuda and Portugal specimens less than 1 mm long, slender, specimens from most other localities comparatively large and bulky; Portuguese specimen was immature. Opaque due to abundance of epidermal glands and oil droplets. Anterior rounded and comparatively slender; mature specimens broaden considerably at level of the statocyst; increase in width more gradual in immature specimens. Posterior tip rounded, possibly with adhesive structure. Frontal glands prominent, opening central, secretion in long bundles; in Bermudian and Portuguese specimens distal base at U35 bulbous, in Adriatic specimens club-shaped (Fig. 8h). Paired testes follicular and lateral; vasa deferentia converge to fsv anterior of mco (Fig. 8g). Male opening in the posterior surrounded by rosette of small glands. Paired ovaries lateral in posterior half or three quarters of body length, oocytes small.

Diagnosis: A common morphological delineation is not possible in this species. Molecular character diagnosis in table 5. Smallest interspecific pairwise distance vs. intraspecific distance 4.8/0.6% (LSU), 5.2/0.3% (SSU) and 14.8/9.9% (H3).

Remarks: This species is supported by the SSU and LSU genes but not by the higher resolving Histone 3 gene. This species is the only truly cosmopolitan species in this dataset. The Histone 3 gene splits the species in geographic clades. This makes the existence of another, not yet resolved species complex, possible. Population genetic studies of this species with increased geographic sampling and sample size might give valuable insights into the dispersal capabilities of interstitial meiofauna.

Distribution: Mediterranean, southern Portugal, Bermuda, New Caledonia, Papua New Guinea, Hawaii.

Etymology: variabilis = latin for variable, as this species is morphologically and genetically changeable throughout its known range.

## Supporting Information

Figure S1 **Saturation plot for the H3 gene across the whole dataset.** Plotted are the uncorrected p-distances versus the phylogenetic distances between pairs of sequences. The level distribution of the points indicates saturation.
(TIFF)

Figure S2 **Best ML tree calculated with RAxML of the LSU rRNA dataset with bootstrap support plotted on the branches.** Putative species with binomial names are formally described in the present study, those with abbreviations represent candidate species. The branch colours correspond to partitions for BP&P analyses, green indicates the *Nemertinoides* group, red the mainly European *Sterreria* subgroup and blue the extra-European *Sterreria* species; orange species have not been validated with BP&P.
(TIFF)

Figure S3 **Majority rule consensus tree estimated with MrBayes of the LSU rRNA dataset with Bayesian posterior probabilities plotted on the nodes.** Putative species with binomial names are formally described in the present study, those with abbreviations represent candidate species. The branch colours correspond to partitions for BP&P analyses, green indicates the *Nemertinoides* group, red the mainly European *Sterreria* subgroup and blue the extra-European *Sterreria* species; orange species have not been validated with BP&P.
(TIFF)

Figure S4 **Best ML tree calculated with RAxML of the SSU rRNA dataset with bootstrap support plotted on the nodes.** Putative species with binomial names are formally described in the present study, those with abbreviations represent candidate species. The branch colours correspond to partitions for BP&P analyses, green indicates the *Nemertinoides* group, red the mainly European *Sterreria* subgroup and blue the extra-European *Sterreria* species; orange species have not been validated with BP&P.
(TIFF)

Figure S5 **Majority rule consensus tree estimated with MrBayes of the SSU rRNA dataset with Bayesian posterior probabilities plotted on the nodes.** Putative species with binomial names are formally described in the present study, those with abbreviations represent candidate species. The branch colours correspond to partitions for BP&P analyses, green indicates the *Nemertinoides* group, red the mainly European *Sterreria* subgroup and blue the extra-European *Sterreria* species; orange species have not been validated with BP&P.
(TIFF)

Figure S6 **Best ML tree calculated with RAxML of the Histone 3 dataset with bootstrap support plotted on the nodes.** Putative species with binomial names are formally described in the present study, those with abbreviations represent candidate species. The branch colours correspond to partitions for BP&P analyses, green indicates the *Nemertinoides* group, red the mainly European *Sterreria* subgroup and blue the extra-European *Sterreria* species; orange species have not been validated with BP&P.
(TIFF)

Figure S7 **Majority rule consensus tree estimated with MrBayes of the Histone 3 dataset with Bayesian posterior probabilities plotted on the nodes.** Putative species with binomial names are formally described in the present study, those with abbreviations represent candidate species. The branch colours correspond to partitions for BP&P analyses, green indicates the *Nemertinoides* group, red the mainly European *Sterreria* subgroup and blue the extra-European *Sterreria* species; orange species have not been validated with BP&P.
(TIFF)

Table S1 **Reduced datasets.** Table showing, which sequences per dataset had been excluded for pairwise distance calculations and TCS analyses.
(XLSX)

Table S2 **Averaged pairwise distances across the LSU gene dataset.**
(XLSX)

Table S3 **Averaged pairwise distances across the SSU gene dataset.**
(XLSX)

Table S4 **Averaged pairwise distances across the Histone 3 gene dataset.** For *Sterreria variabilis* n.sp. pairwise distances are also given for the four subdivisions within the species.
(XLSX)
