# Supplementary figures and images for: Hyper-Cryptic Marine Meiofauna: Species Complexes in Nemertodermatida
Source: PLoS One. 2014 Sep 16;9(9):e107688. doi: 10.1371/journal.pone.0107688 (PMC4166464; doi:10.1371/journal.pone.0107688)

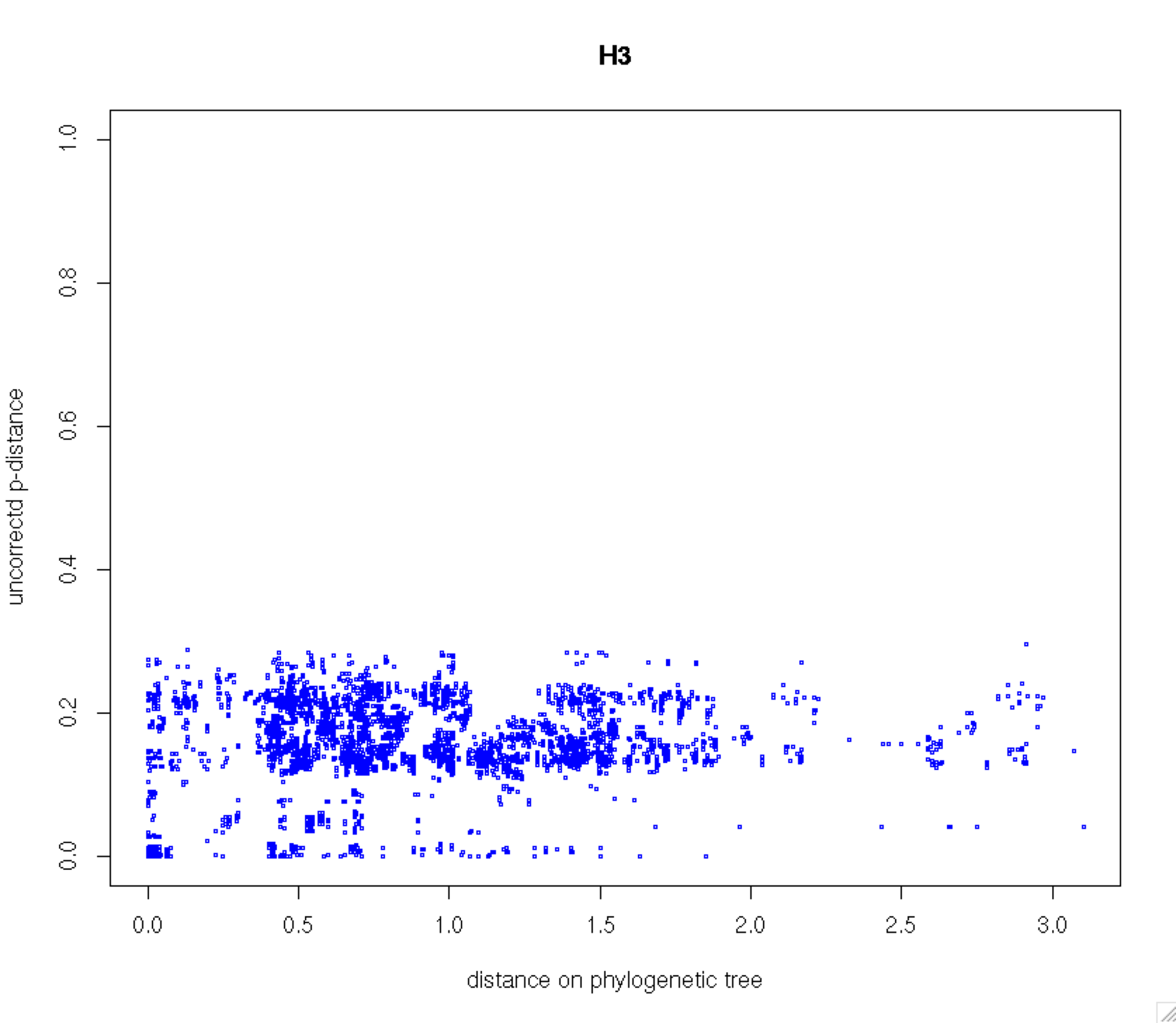

Supplement: Figure S1 — Saturation plot for the H3 gene across the whole dataset. Plotted are the uncorrected p-distances versus the phylogenetic distances between pairs of sequences. The level distribution of the points indicates saturation. (TIFF) [file pone.0107688.s001.tiff]

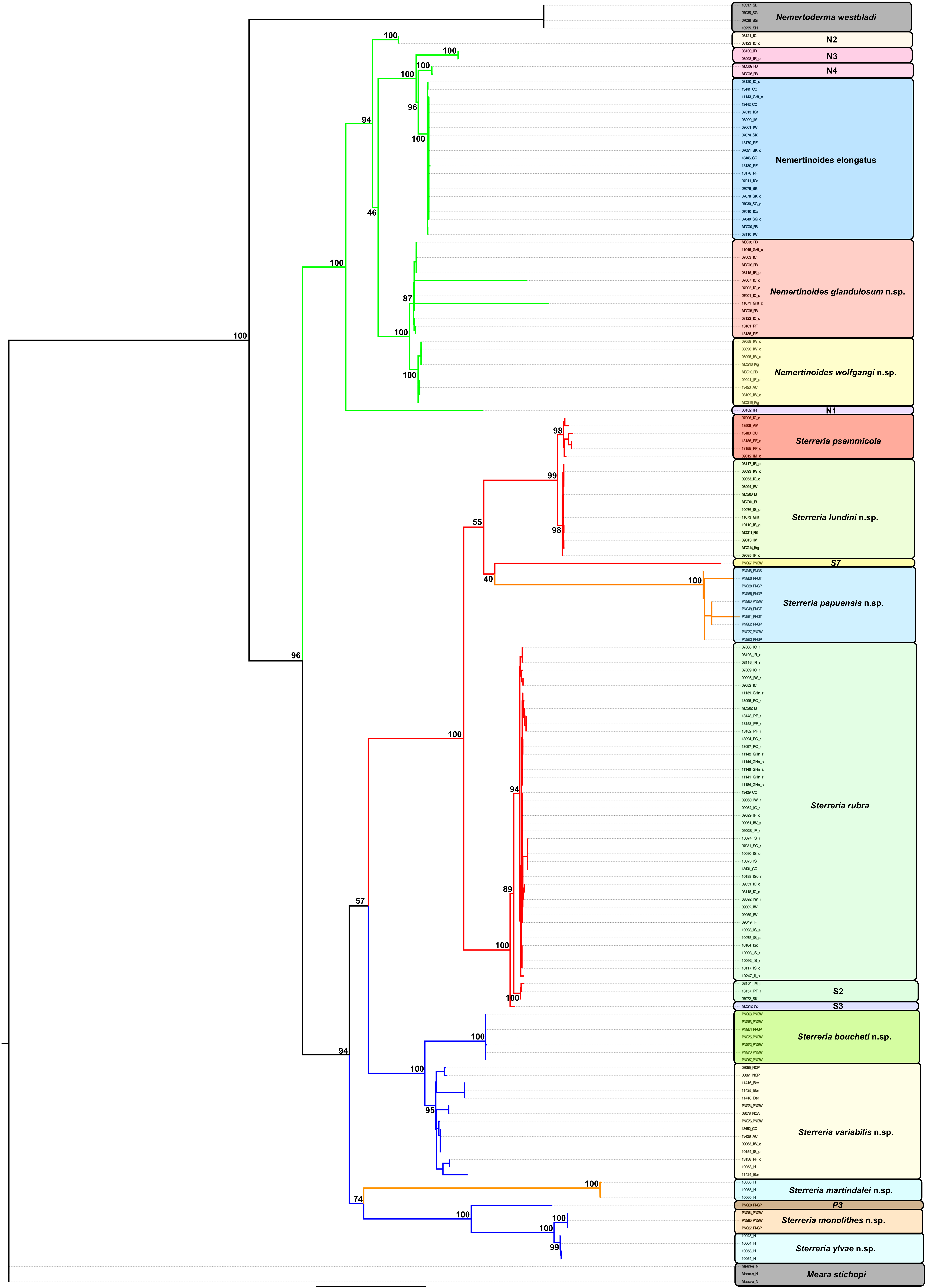

Supplement: Figure S2 — Best ML tree calculated with RAxML of the LSU rRNA dataset with bootstrap support plotted on the branches. Putative species with binomial names are formally described in the present study, those with abbreviations represent candidate species. The branch colours correspond to partitions for BP&P analyses, green indicates the Nemertinoides group, red the mainly European Sterreria subgroup and blue the extra-European Sterreria species; orange species have not been validated with BP&P. (TIFF) [file pone.0107688.s002.tiff]

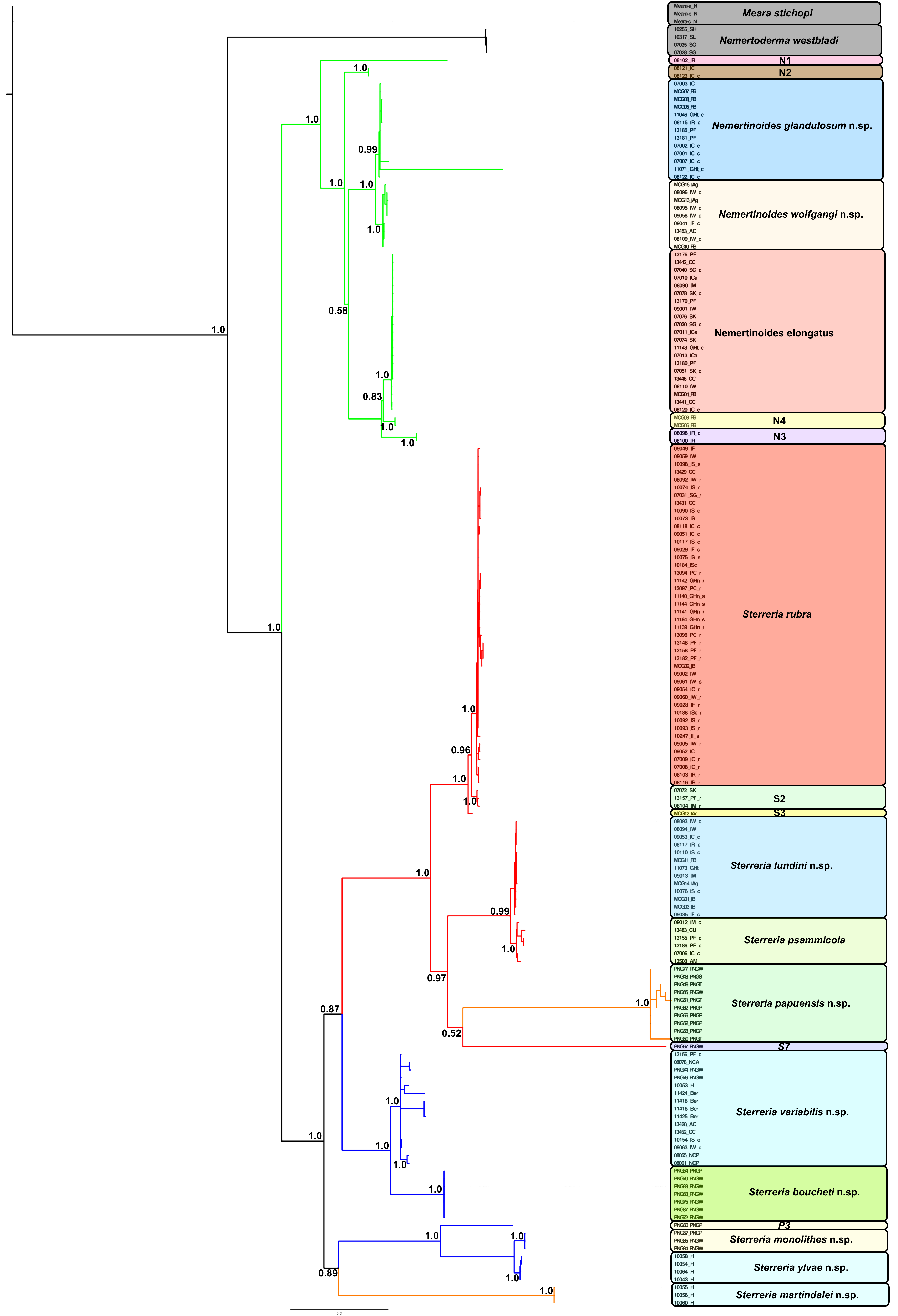

Supplement: Figure S3 — Majority rule consensus tree estimated with MrBayes of the LSU rRNA dataset with Bayesian posterior probabilities plotted on the nodes. Putative species with binomial names are formally described in the present study, those with abbreviations represent candidate species. The branch colours correspond to partitions for BP&P analyses, green indicates the Nemertinoides group, red the mainly European Sterreria subgroup and blue the extra-European Sterreria species; orange species have not been validated with BP&P. (TIFF) [file pone.0107688.s003.tiff]

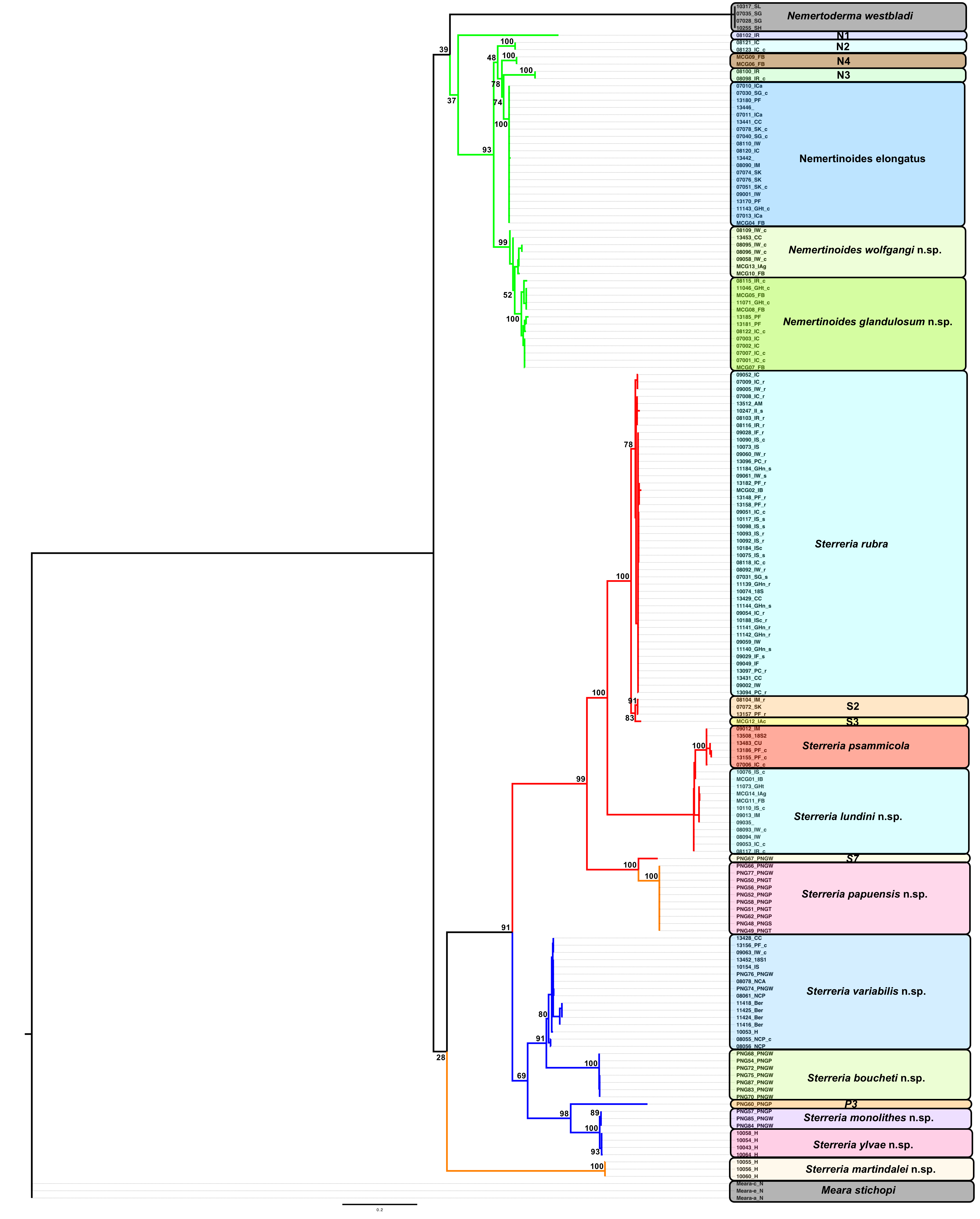

Supplement: Figure S4 — Best ML tree calculated with RAxML of the SSU rRNA dataset with bootstrap support plotted on the nodes. Putative species with binomial names are formally described in the present study, those with abbreviations represent candidate species. The branch colours correspond to partitions for BP&P analyses, green indicates the Nemertinoides group, red the mainly European Sterreria subgroup and blue the extra-European Sterreria species; orange species have not been validated with BP&P. (TIFF) [file pone.0107688.s004.tiff]

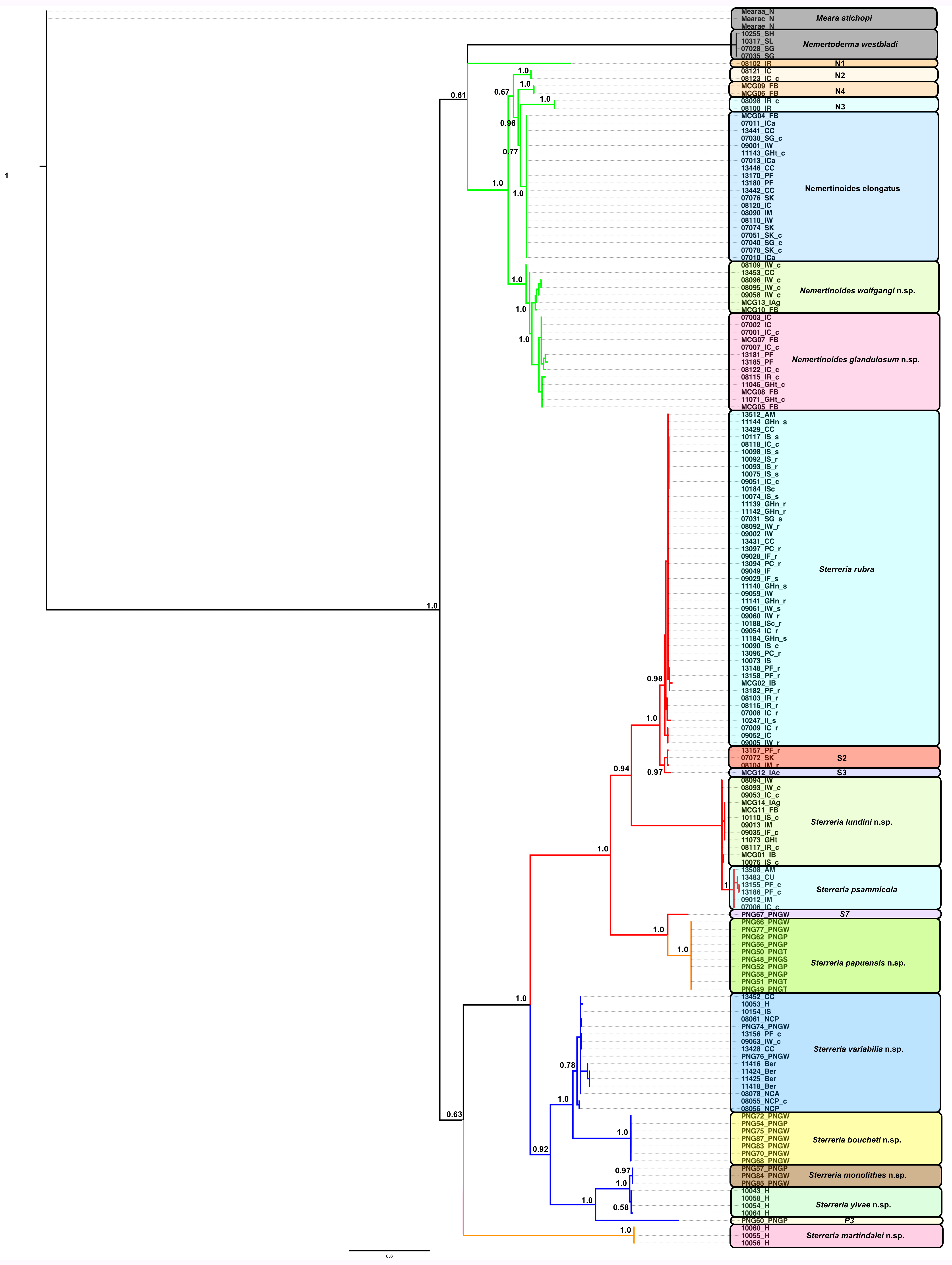

Supplement: Figure S5 — Majority rule consensus tree estimated with MrBayes of the SSU rRNA dataset with Bayesian posterior probabilities plotted on the nodes. Putative species with binomial names are formally described in the present study, those with abbreviations represent candidate species. The branch colours correspond to partitions for BP&P analyses, green indicates the Nemertinoides group, red the mainly European Sterreria subgroup and blue the extra-European Sterreria species; orange species have not been validated with BP&P. (TIFF) [file pone.0107688.s005.tiff]

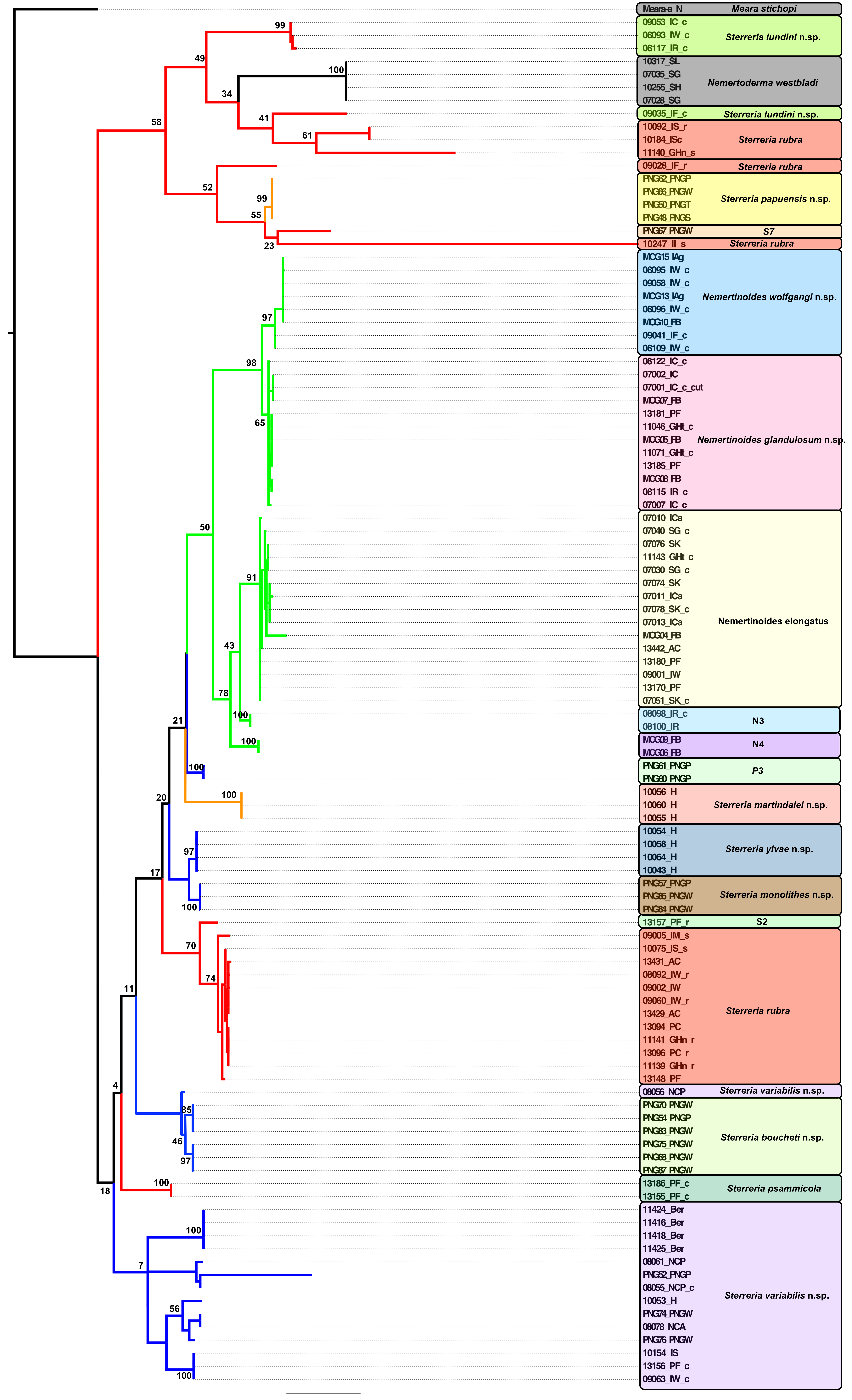

Supplement: Figure S6 — Best ML tree calculated with RAxML of the Histone 3 dataset with bootstrap support plotted on the nodes. Putative species with binomial names are formally described in the present study, those with abbreviations represent candidate species. The branch colours correspond to partitions for BP&P analyses, green indicates the Nemertinoides group, red the mainly European Sterreria subgroup and blue the extra-European Sterreria species; orange species have not been validated with BP&P. (TIFF) [file pone.0107688.s006.tiff]

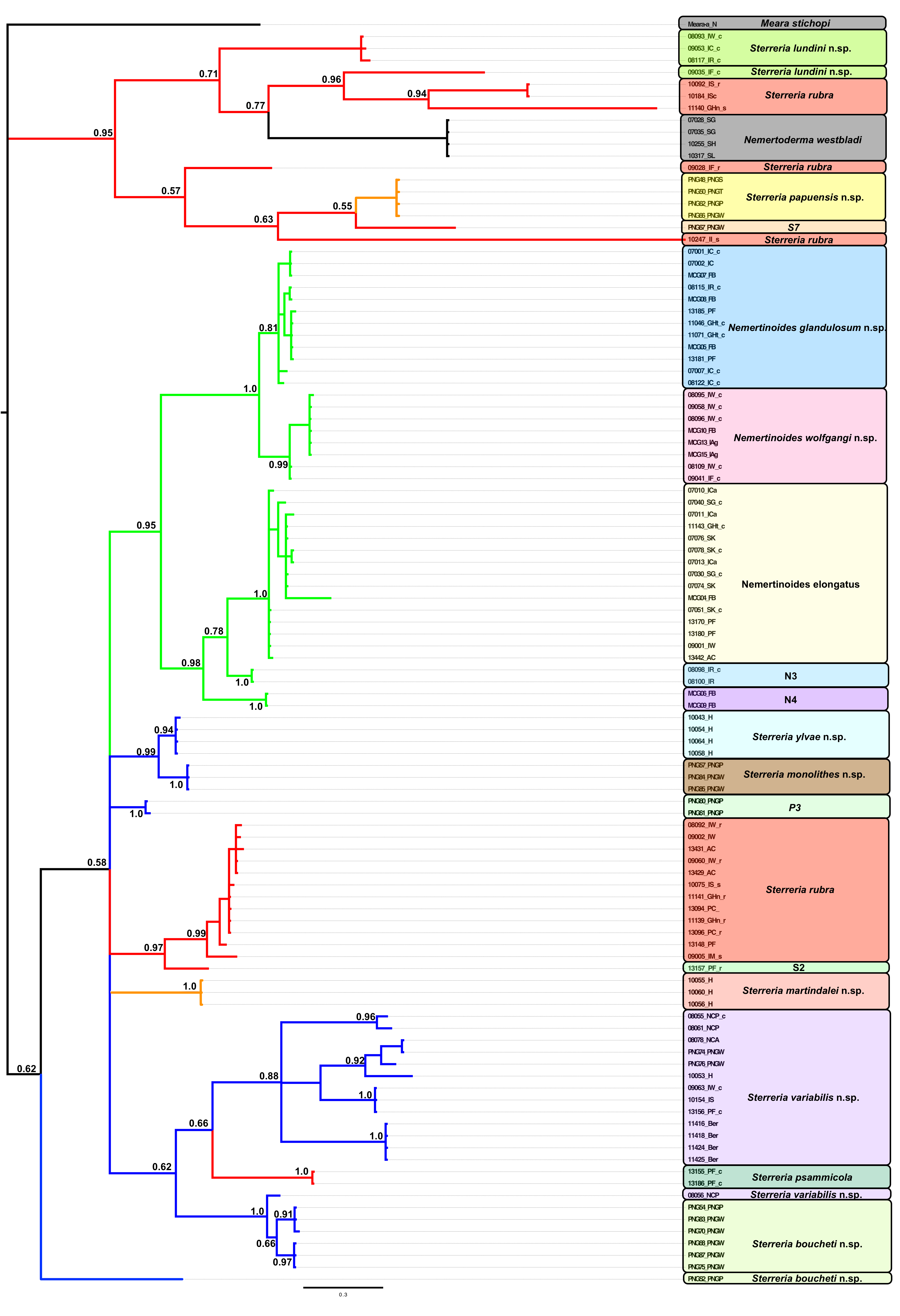

Supplement: Figure S7 — Majority rule consensus tree estimated with MrBayes of the Histone 3 dataset with Bayesian posterior probabilities plotted on the nodes. Putative species with binomial names are formally described in the present study, those with abbreviations represent candidate species. The branch colours correspond to partitions for BP&P analyses, green indicates the Nemertinoides group, red the mainly European Sterreria subgroup and blue the extra-European Sterreria species; orange species have not been validated with BP&P. (TIFF) [file pone.0107688.s007.tiff]
